# Supplementary material for: Soil degradation and herbicide pollution by repeated cassava monoculture within Thailand’s conservation region
Source: PLoS One. 2024 Aug 6;19(8):e0308284. doi: 10.1371/journal.pone.0308284 (PMC11302848; doi:10.1371/journal.pone.0308284)
Supplement: S1 Fig — (A) Kjeldahl nitrogen (the sum of organic N and ammonia N; g kg-1), (B) available phosphorus (P; mg kg-1), (C) exchangeable potassium (K; mg kg-1), (D) organic carbon (g kg-1), (E) pH, (F) electrical conductivity (EC; mS m-1), (G) cation exchange capacity (cmol(+) kg-1), (H) fraction of sand (%), (I) fraction of silt (%), (J) fraction of clay (%), (K) bulk density (Mg m-3), and (L) gravimetric moisture content (%). The boxplot shows the median (horizontal bar), interquartile range (box), 5th and 95th percentiles (whiskers; vertical bars), and outliers (black closed points). Different letters (a, b, c) show the significant differences among the years, as follows: ***: P < 0.001, **: P < 0.01, *: P < 0.05, and n.s.: P ≥ 0.05. (PDF) [file pone.0308284.s004.pdf]

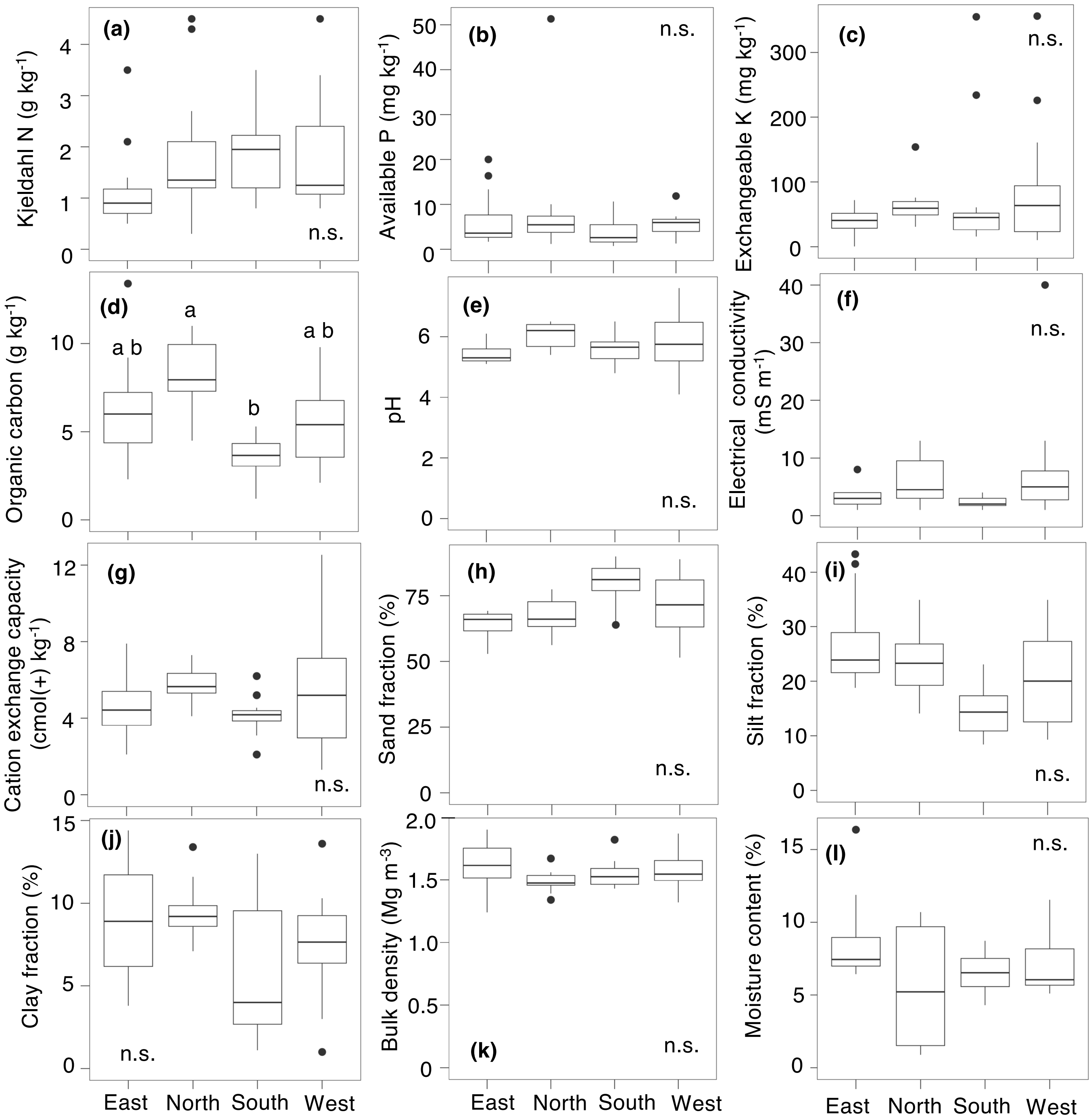

S1 Fig. Variations in soil properties among the locations in the farmland. (**A**) Kjeldahl nitrogen (the sum of organic N and ammonia N; g kg^{-1}), (**B**) available phosphorus (P; mg kg^{-1}), (**C**) exchangeable potassium (K; mg kg^{-1}), (**D**) organic carbon (g kg^{-1}), (**E**) pH, (**F**) electrical conductivity (EC; mS m^{-1}), (**G**) cation exchange capacity (cmol(+) kg^{-1}), (**H**) fraction of sand (%), (**I**) fraction of silt (%), (**J**) fraction of clay (%), (**K**) bulk density (g cm^{-3}), and (**L**) gravimetric moisture content (%). The boxplot shows the median (horizontal bar), interquartile range (box), 5th and 95th percentiles (whiskers; vertical bars), and outliers (black closed points). Different letters (a, b) show the significant differences ($P < 0.05$) among the locations, and "n.s." means no significant differences.
