# Supplementary material for: Soil degradation and herbicide pollution by repeated cassava monoculture within Thailand’s conservation region
Source: PLoS One. 2024 Aug 6;19(8):e0308284. doi: 10.1371/journal.pone.0308284 (PMC11302848; doi:10.1371/journal.pone.0308284)
Supplement: Attachment — Submitted filename: General comments.pdf [file pone.0308284.s005.pdf]

General comments

The study sought to investigate the impacts of cassava monoculture on soil properties and the effects of herbicide application on farmland soils in Thailand. Though the manuscript could be beneficial to the readership of the journal as it elucidates the impact of monoculture on soil fertility variables, it lacks clarity and novelty.

The discussion section of the manuscript is poorly written. The authors largely failed to discuss the results obtained from the study, and this makes the manuscript technically bankrupt to be accepted for publication in its current form.

Below are my specific comments:

Line 89: change "import" to "importation"

Lines 118 – 141: The section should be summarized and moved to the Materials and Methods section of the manuscript.

What is/ are the objective(s) of the study? This should be clearly stated in the introduction section of the manuscript. Also, state the hypothesis/ hypothesis that was/ were tested.

Line 161: Change "Material sampling" to "Soil and water sampling".

Lines 190 – 191: The import of this sentence is that you equally determined the soil bulk density with the hydrometer method which is not correct. Please rephrase the sentence to make it scientifically correct.

How was the soil bulk density determined?

Lines 197 – 199: This has already been stated in Lines 184 – 186. Please delete.

Line 234: "using"

Lines 256 – 258: Is it part of the statistical analysis? Please delete.

Lines 270 – 271: I am sure you meant to state that the average concentrations of N, P, and K in the farmland soil were 1.9 times, 3.1 times, and 6.3 times **higher than** those in the forest soil, respectively. Please rephrase the sentence to make it comprehensible.

Lines 300 – 317: This section could be moved to the introduction. You are to discuss the results obtained from the study and not to highlight on general literature.

Lines 321 – 323: Run a simple correlation analysis to substantiate this claim. The cation exchange capacity can equally be affected by the soil pH and organic matter. Authors should correlate the

measured soil variables with CEC. Simple correlation coefficients could be used to enrich their discussion.

Line 331 – 334: Rephrase the sentence to make it coherent.

Lines 365 – 442: You did not discuss the results obtained. In a nutshell, the discussion was poorly written. The discussion section needs to be revised.

In conclusion, what are the implications of the findings of the study?
