# Supplementary material for: Soil degradation and herbicide pollution by repeated cassava monoculture within Thailand’s conservation region
Source: PLoS One. 2024 Aug 6;19(8):e0308284. doi: 10.1371/journal.pone.0308284 (PMC11302848; doi:10.1371/journal.pone.0308284)
Supplement: Attachment — Submitted filename: PONE-D-24-04738_R1 (2).pdf [file pone.0308284.s006.pdf]

PLOS ONE

Soil degradation and herbicide pollution by repeated cassava monoculture within Thailand's conservation region

--Manuscript Draft--

**Manuscript Number:**	PONE-D-24-04738R1
**Article Type:**	Research Article
**Full Title:**	Soil degradation and herbicide pollution by repeated cassava monoculture within Thailand's conservation region
**Short Title:**	Soil degradation and herbicide pollution in cassava monoculture
**Corresponding Author:**	Atsushi Ishida<br>Kyoto University<br>Otsu, Shiga JAPAN
**Keywords:**	cassava, monoculture, glyphosate, soil degradation, tropical forest reserve
**Abstract:**	In a national park in Northeast Thailand, agricultural land has been converted from natural forest by small-scale farmers for cassava agriculture. We hypothesise that long-termed cassava monoculture leads to soil degradation. To test the hypothesis, we conducted a five-year (2016-2020) study on the physical and chemical properties of soil in cassava farmland, and also examined the soil properties of its adjacent natural forests, as a control. Soil nitrogen, available phosphate and potassium, and bulk density were significantly higher in the farmland than in the forest, whereas cation exchange capacity, organic carbon, and clay fraction showed the opposite trend. The cation exchange capacity was positively correlated to the fraction of clay and the organic carbon within soil. These data indicate that the farmland soil was irreversibly degraded by the conversion from forest to cassava fields, through rapid decreases in cation exchange capacity, organic carbon, and the fraction of clay. For the farmland, soil nitrogen and bulk density increased significantly, whereas pH and organic carbon decreased significantly over the five years, indicating that these soil properties were gradually altered by repeated agricultural activities, such as fertilizer application and trampling. The use of glyphosate and paraquat herbicides is prohibited within national parks in Thailand. However, in 2020, glyphosate was detected in farmland soil (up to 5.0 mg kg-1) during both the rainy and dry seasons, and glyphosate (2.5 mg l-1) was detected in stream water from the farmland during the dry season. The use of glyphosate in the farmlands was thus suspected at least in 2020. Soil degradation and herbicide pollution may carry a high risk of causing irreversible changes in terrestrial ecosystems. We discuss the root causes of this issue from perspectives of agricultural production, economy, and the environmental impact, and propose effective policy measures.
**Order of Authors:**	Atsushi Ishida
	Ananya Popradit
	Yutthana Nakhokwik
	Marcel Robischon
	Shin-Taro Saiki,
	Jin Yoshimura
	Arichai Wanasiri
**Opposed Reviewers:**	
**Response to Reviewers:**	To Editor,<br><br>Thank you for your support for publishing in PLoS ONE. We carefully read the comments from the editor and reviewers, and revised MS for all points. In the attached "Tracking_file", I show the added or changed parts with yellow-color highlight.<br>We revised the abstract, and added some information in the Material & Methods. We added two new figures (Figs 2 and 4) for enhancing the visualization of the obtained

Powered by Editorial Manager® and ProduXion Manager® from Aries Systems Corporation

data. Moreover, we reconsidered and changed the statical approach for data analysis, especially in the multiple comparison (Table 2) and in the correlation analysis related to the cation exchange capacity (Fig 4 and Table 4).
In the revised MS, we added the hypothesis in the abstract (Line 50-51) and the introduction (Line 126-127). In the discussion part, I newly added discussion related to soil properties (Line 318-370) and the conclusion in the last part of discussion (Line 485-500).
According to the comments, we largely revised the MS. We believe that we are able to enhance the scientific validity and the visualization of the obtained data. We clearly show the key findings in the conclusion. Thank you very much for valuable comments from the editor and reviewers.

Atsushi Ishida, the corresponding author

Reviewers' comments:

Reviewer #1: General comments

The study sought to investigate the impacts of cassava monoculture on soil properties and the effects of herbicide application on farmland soils in Thailand. Though the manuscript could be beneficial to the readership of the journal as it elucidates the impact of monoculture on soil fertility variables, it lacks clarity and novelty.
The discussion section of the manuscript is poorly written. The authors largely failed to discuss the results obtained from the study, and this makes the manuscript technically bankrupt to be accepted for publication in its current form.

Reply: Thank you for your support for publishing in PLoS ONE. We carefully read the comments from the editor and reviewers., and revised MS for all points. In the attached “Tracking_file”, I show the added or changed parts with yellow-color highlight.
We revised the abstract, and added some information in the Material & Methods. We added two figures (Figs 2 and 4). We reconsidered and changed the statical approach for data analysis. In the Discussion part, I added new discussion related to soil properties and added the conclusion in the last part of discussion.
According to the comments, we largely revised the MS. We believe that we are able to enhance the scientific validity and the visualization of the obtained data. Thank you very much for your valuable comments.

Below are my specific comments:
Line 89: change “import” to “importation”
Reply: I changed it.

Lines 118 – 141: The section should be summarized and moved to the Materials and Methods section of the manuscript.
What is/ are the objective(s) of the study? This should be clearly stated in the introduction section of the manuscript. Also, state the hypothesis/ hypothesis that was/ were tested.
Reply: We moved this part to the head of Material and Methods as a method overview (Line 141-165). In the revised MS, we added the hypothesis in Abstract (Line 50-51) and Introduction (Line 126-127).

Line 161: Change “Material sampling” to “Soil and water sampling”.
Reply: We changed it.

Lines 190 – 191: The import of this sentence is that you equally determined the soil bulk density with the hydrometer method which is not correct. Please rephrase the sentence to make it scientifically correct. How was the soil bulk density determined?
Reply: Sorry, we mistook the statements. We revised the parts in the material and methods (Line 208-211)

Lines 197 – 199: This has already been stated in Lines 184 – 186. Please delete.
Reply: We deleted it.

Line 234: “using”

Reply: We changed it.

Lines 256 – 258: Is it part of the statistical analysis? Please delete.
Reply: We moved this part from the statistical analysis to the first of the results (Line 270-271).

Lines 270 – 271: I am sure you meant to state that the average concentrations of N, P, and K in the farmland soil were 1.9 times, 3.1 times, and 6.3 times higher than those in the forest soil, respectively. Please rephrase the sentence to make it comprehensible.
Reply: We rephrased it (Line 281-282).

Lines 300 – 317: This section could be moved to the introduction. You are to discuss the results obtained from the study and not to highlight on general literature.
Reply: In the revised MS, we moved this part to the introduction from the discussion (Line 103-111).

Lines 321 – 323: Run a simple correlation analysis to substantiate this claim. The cation exchange capacity can equally be affected by the soil pH and organic matter. Authors should correlate the measured soil variables with CEC. Simple correlation coefficients could be used to enrich their discussion.
Reply: We want to say very thank you for this important suggestion. We added new figure of the correlation analyses for examining the effects of cation exchange on the clay fraction, the organic carbon, and pH (Fig 4). Furthermore, we added a multiple correlation analysis related to Fig 4 (Table 4). According to this analysis and your suggestion, we were able to add significant discussion related to soil degradation (Line 318-370).

Line 331 – 334: Rephrase the sentence to make it coherent.
Reply: We rephrased it (Line 281-282).

Lines 365 – 442: You did not discuss the results obtained. In a nutshell, the discussion was poorly written. The discussion section needs to be revised.
In conclusion, what are the implications of the findings of the study?
Reply: In the Discussion part, we added new discussion related to soil properties (Line 318-370). In the last part of discussion, we added the conclusion to clarify the key findings (Line 485-500).

Atsushi Ishida, the corresponding author

Reviewers' comments:
Reviewer #2:

The description of weather conditions is not sufficient (lines 147-150). To justify changes in soil conditions, it is necessary to provide a complete description of climatic conditions over the years of research.
In the “Results” section, the authors refer to different seasons (wet and dry), but the years are not specified.
The tables do not indicate the years or period the data was obtained.
The “Conclusions” section is missing. It must be written and inserted into the article. It is necessary to review the list of references and bring them by the writing rules.

Reply: Thank you for your support for publishing in PLoS ONE. We carefully read all comments from the editor and reviewers, and revised MS for all points. In the attached “Tracking_file”, we show the added or changed parts with yellow-color highlight.
We agree with your suggestion that climate data is important. We added climatological data in Fig 1(D). In the annual changes in farmland soil properties, we added the multiple comparisons between the years and the locations, using Tukey’s honestly significant difference (HSD) test (Table 3). In Table 3, more detailed information (including years) is shown.
We added the conclusion in the last part of the discussion to clarify the key findings (Line 485-500).
We adjusted the reference list, according to the rule of PLoS ONE.

Powered by Editorial Manager® and ProduXion Manager® from Aries Systems Corporation

	Atsushi Ishida, the corresponding author
**Additional Information:**	
**Question**	**Response**
**Financial Disclosure**<br><br>Enter a financial disclosure statement that describes the sources of funding for the work included in this submission. Review the submission guidelines for detailed requirements. View published research articles from *PLOS ONE* for specific examples.<br><br>This statement is required for submission and **will appear in the published article** if the submission is accepted. Please make sure it is accurate.<br><br>**Funded studies**<br>Enter a statement with the following details:<br>• Initials of the authors who received each award<br>• Grant numbers awarded to each author<br>• The full name of each funder<br>• URL of each funder website<br>• Did the sponsors or funders play any role in the study design, data collection and analysis, decision to publish, or preparation of the manuscript?<br><br>Did you receive funding for this work?	Yes
Please add funding details.<br>as follow-up to "**Financial Disclosure**<br><br>Enter a financial disclosure statement that describes the sources of funding for the work included in this submission. Review the submission guidelines for detailed requirements. View published research articles from *PLOS ONE* for specific examples.<br><br>This statement is required for submission and **will appear in the published article** if the submission is accepted. Please make sure it is accurate.	This study was supported by funding provided from the Japan Society for the Promotion of Science (No. 16H02708, 23KK0119, 24K03129). The funders had no role in study design, data collection and analysis, decision to publish, or preparation of the manuscript.

Powered by Editorial Manager® and ProduXion Manager® from Aries Systems Corporation

**Funded studies**<br>Enter a statement with the following details:<br>• Initials of the authors who received each award<br>• Grant numbers awarded to each author<br>• The full name of each funder<br>• URL of each funder website<br>• Did the sponsors or funders play any role in the study design, data collection and analysis, decision to publish, or preparation of the manuscript?<br><br>Did you receive funding for this work?"	
Please select the country of your main research funder (please select carefully as in some cases this is used in fee calculation).<br>as follow-up to "**Financial Disclosure**<br><br>Enter a financial disclosure statement that describes the sources of funding for the work included in this submission. Review the submission guidelines for detailed requirements. View published research articles from *PLOS ONE* for specific examples.<br><br>This statement is required for submission and **will appear in the published article** if the submission is accepted. Please make sure it is accurate.<br><br>**Funded studies**<br>Enter a statement with the following details:<br>• Initials of the authors who received each award<br>• Grant numbers awarded to each author<br>• The full name of each funder<br>• URL of each funder website<br>• Did the sponsors or funders play any role in the study design, data collection and analysis, decision to publish, or preparation of the manuscript?<br><br>Did you receive funding for this work?"	JAPAN - JP

**Competing Interests**<br><br>Use the instructions below to enter a competing interest statement for this submission. On behalf of all authors, disclose any competing interests that could be perceived to bias this work—acknowledging all financial support and any other relevant financial or non-financial competing interests.<br><br>This statement is required for submission and **will appear in the published article** if the submission is accepted. Please make sure it is accurate and that any funding sources listed in your Funding Information later in the submission form are also declared in your Financial Disclosure statement.<br><br>View published research articles from *PLOS ONE* for specific examples.<br><br>**NO authors have competing interests**<br><br>Enter: *The authors have declared that no competing interests exist.*<br><br>**Authors with competing interests**<br><br>Enter competing interest details beginning with this statement:<br><br>*I have read the journal's policy and the authors of this manuscript have the following competing interests: [insert competing interests here]*<br><br>* typeset	The authors declare no competing interests.
**Ethics Statement**<br><br>Enter an ethics statement for this submission. This statement is required if the study involved:<br><br>• Human participants<br>• Human specimens or tissue<br>• Vertebrate animals or cephalopods	This field study was approved by the Department of National Parks, Wildlife and Plant Conservation in the Thai government (Permission No. 5810701, 5810107, 5921601). All procedures in this study were carried out in accordance with the relevant guidelines of Valaya Alongkorn Rajabhat University.

Powered by Editorial Manager® and ProduXion Manager® from Aries Systems Corporation

- Vertebrate embryos or tissues
- Field research

Write "N/A" if the submission does not require an ethics statement.

General guidance is provided below. Consult the submission guidelines for detailed instructions. **Make sure that all information entered here is included in the Methods section of the manuscript.**

Powered by Editorial Manager® and ProduXion Manager® from Aries Systems Corporation

**Format for specific study types**<br><br>**Human Subject Research (involving human participants and/or tissue)**<br>• Give the name of the institutional review board or ethics committee that approved the study<br>• Include the approval number and/or a statement indicating approval of this research<br>• Indicate the form of consent obtained (written/oral) or the reason that consent was not obtained (e.g. the data were analyzed anonymously)<br><br>**Animal Research (involving vertebrate animals, embryos or tissues)**<br>• Provide the name of the Institutional Animal Care and Use Committee (IACUC) or other relevant ethics board that reviewed the study protocol, and indicate whether they approved this research or granted a formal waiver of ethical approval<br>• Include an approval number if one was obtained<br>• If the study involved *non-human primates*, add *additional details* about animal welfare and steps taken to ameliorate suffering<br>• If anesthesia, euthanasia, or any kind of animal sacrifice is part of the study, include briefly which substances and/or methods were applied<br><br>**Field Research**<br><br>Include the following details if this study involves the collection of plant, animal, or other materials from a natural setting:<br>• Field permit number<br>• Name of the institution or relevant body that granted permission	
**Data Availability**<br><br>Authors are required to make all data underlying the findings described fully available, without restriction, and from the time of publication. PLOS allows rare exceptions to address legal and ethical concerns. See the PLOS Data Policy and FAQ for detailed information.	Yes - all data are fully available without restriction

A Data Availability Statement describing where the data can be found is required at submission. Your answers to this question constitute the Data Availability Statement and **will be published in the article**, if accepted.<br><br>**Important:** Stating 'data available on request from the author' is not sufficient. If your data are only available upon request, select 'No' for the first question and explain your exceptional situation in the text box.<br><br>Do the authors confirm that all data underlying the findings described in their manuscript are fully available without restriction?	
**Describe where the data may be found in full sentences. If you are copying our sample text, replace any instances of XXX with the appropriate details.**<br><br>• If the data are **held or will be held in a public repository**, include URLs, accession numbers or DOIs. If this information will only be available after acceptance, indicate this by ticking the box below. For example: *All XXX files are available from the XXX database (accession number(s) XXX, XXX.)*.<br>• If the data are all contained **within the manuscript and/or Supporting Information files**, enter the following: *All relevant data are within the manuscript and its Supporting Information files.*<br>• If neither of these applies but you are able to provide **details of access elsewhere**, with or without limitations, please do so. For example:<br><br>*Data cannot be shared publicly because of [XXX]. Data are available from the XXX Institutional Data Access / Ethics Committee (contact via XXX) for researchers who meet the criteria for access to confidential data.*<br><br>*The data underlying the results presented in the study are available from (include the name of the third party*	All raw data are within the paper and its Supporting Information files.

and contact information or URL).<br>• This text is appropriate if the data are owned by a third party and authors do not have permission to share the data.<br><br>* typeset	
Additional data availability information:	

Powered by Editorial Manager® and ProduXion Manager® from Aries Systems Corporation

PONE-D-24-04738
Soil degradation and herbicide pollution by repeated cassava monoculture within Thailand’s conservation region

Additional Editor Comments:

Based on reviewers' comments and my evaluation, the authors need to conduct a comprehensive and detailed revision to meet the standards required for further review. Specifically, attention should be given to the following aspects:
The abstract should be focused and clear, providing a succinct summary of the study's objectives, methods, key findings, and implications. The sampling procedure needs to be elaborated upon with greater detail, including the specific name of the sampling method employed. This will enhance transparency and reproducibility. The statistical analyses, particularly those based on ANOVAs, should be thoroughly explained, including the interactions between years and locations. Clear justification for the choice of statistical methods is essential for robust interpretation of results. It is advisable for the authors to seek guidance from a biostatistician to ensure proper organization, analysis, and presentation of the data in tables and figures. This collaboration will enhance the accuracy and validity of the statistical analyses.

Based on appropriate statistical analysis, the results should be presented in tables and figures, accompanied by concise explanations to facilitate understanding and interpretation. Optimization of Figure Scales: Figures should be optimized to ensure clear visualization of the data, with appropriate scaling to accurately represent the findings. A conclusive section summarizing the key findings, implications, and potential avenues for future research should be added to provide closure to the manuscript.

Overall, the manuscript requires significant improvements to meet the standards for publication. By addressing the aforementioned areas and collaborating with a biostatistician, the authors can enhance the scientific rigor, credibility, and relevance of their study, thereby attracting the attention of a wider international audience in the field of soil science.

To Editor,

Thank you for your support for publishing in PLoS ONE. We carefully read the comments from the editor and reviewers, and revised MS for all points. In the attached "Tracking_file", I show the added or changed parts with yellow-color highlight.

We revised the abstract, and added some information in the Material & Methods. We added two new figures (Figs 2 and 4) for enhancing the visualization of the obtained data. Moreover, we reconsidered and changed the statical approach for data analysis, especially in the multiple comparison (Table 2) and in the correlation analysis related to the cation exchange capacity (Fig 4 and Table 4).

In the revised MS, we added the hypothesis in the abstract (Line 50-51) and the introduction (Line 126-127). In the discussion part, I newly added discussion related to soil properties (Line 318-370) and the conclusion in the last part of discussion (Line 485-500).

According to the comments, we largely revised the MS. We believe that we are able to enhance the scientific validity and the visualization of the obtained data. We clearly show the key findings in the conclusion. Thank you very much for valuable comments from the editor and reviewers.

Atsushi Ishida, the corresponding author

PONE-D-24-04738

Soil degradation and herbicide pollution by repeated cassava monoculture within Thailand's conservation region

Reviewers' comments:

Reviewer #1: General comments

The study sought to investigate the impacts of cassava monoculture on soil properties and the effects of herbicide application on farmland soils in Thailand. Though the manuscript could be beneficial to the readership of the journal as it elucidates the impact of monoculture on soil fertility variables, it lacks clarity and novelty.

The discussion section of the manuscript is poorly written. The authors largely failed to discuss the results obtained from the study, and this makes the manuscript technically bankrupt to be accepted for publication in its current form.

Reply: Thank you for your support for publishing in PLoS ONE. We carefully read the comments from the editor and reviewers., and revised MS for all points. In the attached "Tracking_file", I show the added or changed parts with yellow-color highlight.

We revised the abstract, and added some information in the Material & Methods. We added two figures (Figs 2 and 4). We reconsidered and changed the statical approach for data analysis. In the Discussion part, I added new discussion related to soil properties and added the conclusion in the last part of discussion.

According to the comments, we largely revised the MS. We believe that we are able to enhance the scientific validity and the visualization of the obtained data. Thank you very much for your valuable comments.

Below are my specific comments:

Line 89: change "import" to "importation"

Reply: I changed it.

Lines 118 – 141: The section should be summarized and moved to the Materials and Methods section of the manuscript.

What is/ are the objective(s) of the study? This should be clearly stated in the introduction section of the manuscript. Also, state the hypothesis/ hypothesis that was/ were tested.

Reply: We moved this part to the head of Material and Methods as a method overview (Line 141-165). In the revised MS, we added the hypothesis in Abstract (Line 50-51) and Introduction (Line 126-127).

Line 161: Change “Material sampling” to “Soil and water sampling”.

Reply: We changed it.

Lines 190 – 191: The import of this sentence is that you equally determined the soil bulk density with the hydrometer method which is not correct. Please rephrase the sentence to make it scientifically correct. How was the soil bulk density determined?

Reply: Sorry, we mistook the statements. We revised the parts in the material and methods (Line 208-211)

Lines 197 – 199: This has already been stated in Lines 184 – 186. Please delete.

Reply: We deleted it.

Line 234: “using”

Reply: We changed it.

Lines 256 – 258: Is it part of the statistical analysis? Please delete.

Reply: We moved this part from the statistical analysis to the first of the results (Line 270-271).

Lines 270 – 271: I am sure you meant to state that the average concentrations of N, P, and K in the farmland soil were 1.9 times, 3.1 times, and 6.3 times higher than those in the forest soil, respectively. Please rephrase the sentence to make it comprehensible.

Reply: We rephrased it (Line 281-282).

Lines 300 – 317: This section could be moved to the introduction. You are to discuss the results obtained from the study and not to highlight on general literature.

Reply: In the revised MS, we moved this part to the introduction from the discussion (Line 103-111).

Lines 321 – 323: Run a simple correlation analysis to substantiate this claim. The cation exchange capacity can equally be affected by the soil pH and organic matter. Authors should correlate the measured soil variables with CEC. Simple correlation coefficients could be used to enrich their discussion.

Reply: We want to say very thank you for this important suggestion. We added new figure of the correlation analyses for examining the effects of cation exchange on the clay fraction, the organic carbon, and pH (Fig 4). Furthermore, we added a multiple correlation analysis related to Fig 4 (Table 4). According to this analysis and your suggestion, we were able to add significant discussion related to soil degradation (Line 318-370).

Line 331 – 334: Rephrase the sentence to make it coherent.

Reply: We rephrased it (Line 281-282).

Lines 365 – 442: You did not discuss the results obtained. In a nutshell, the discussion was poorly written. The discussion section needs to be revised.
In conclusion, what are the implications of the findings of the study?

Reply: In the Discussion part, we added new discussion related to soil properties (Line 318-370). In the last part of discussion, we added the conclusion to clarify the key findings (Line 485-500).

Atsushi Ishida, the corresponding author

Reviewers' comments:

Reviewer #2:

The description of weather conditions is not sufficient (lines 147-150). To justify changes in soil conditions, it is necessary to provide a complete description of climatic conditions over the years of research.
In the “Results” section, the authors refer to different seasons (wet and dry), but the years are not specified.
The tables do not indicate the years or period the data was obtained.
The “Conclusions” section is missing. It must be written and inserted into the article.
It is necessary to review the list of references and bring them by the writing rules.

Reply: Thank you for your support for publishing in PLoS ONE. We carefully read all comments from the editor and reviewers, and revised MS for all points. In the attached “Tracking_file”, we show the added or changed parts with yellow-color highlight.

We agree with your suggestion that climate data is important. We added climatological data in Fig 1(D). In the annual changes in farmland soil properties, we added the multiple comparisons between the years and the locations, using Tukey’s honestly significant difference (HSD) test (Table 3). In Table 3, more detailed information (including years) is shown.

We added the conclusion in the last part of the discussion to clarify the key findings (Line 485-500).

We adjusted the reference list, according to the rule of PLoS ONE.

Atsushi Ishida, the corresponding author

Manuscript

Click here to access/download;Manuscript;MainText_Ishida.docx

Title: Soil degradation and herbicide pollution by repeated cassava monoculture within Thailand's conservation region

Short Title: Soil degradation and herbicide pollution in cassava monoculture

Authors: Ananya Popradit (ORCID 0000-0002-1546-7813) [1*], Yutthana Nakhokwik[1], Marcel Robischon[2], Shin-Taro Saiki (ORCID 0000-0002-3685-2665) [3], Jin Yoshimura (ORCID 0000-0003-1610-1386) [4,5,6], Arichai Wanasiri[7] and Atsushi Ishida (ORCID 0000-0003-0620-5819) [8*]

Affiliations:

1 Doctor of Philosophy Program in Environmental Studies, Valaya Alongkorn Rajabhat University under Royal patronage, Pathum Thani 13180, Thailand.

2 Faculty of Life Sciences, Albrecht Daniel Thaer Institute for Agricultural and Horticultural Sciences, Department of Agroecology, Humboldt University of Berlin, Berlin D-10099, Germany.

3 Forestry and Forest Products Research Institute, Matsuno-sato, Tsukuba, Ibaraki 305-8687, Japan.

4 Institute of Tropical Medicine, Nagasaki University, Sakamoto, Nagasaki, Nagasaki 852-8523, Japan.

5 Faculty of Science, Tokyo Metropolitan University, Minami-Osawa, Hachioji, Tokyo 192-0397, Japan.

6 The University Museum, The University of Tokyo, Hongo, Bunkyo, Tokyo 113-0033, Japan.

7 Department of National Parks, Wildlife and Plant Conservation. Ban Kho Subdistrict, Non Sang District, Nong Bua Lamphu 39140, Thailand.

8 Center for Ecological Research, Kyoto University, Otsu, Shiga 520-2113, Japan.

Correspondence Authors:

Ananya Popradit: ananya-toon@hotmail.co.th

Atsushi Ishida: atto@ecology.kyoto-u.ac.jp

Data Availability Statement: All raw data are within the paper and its Supporting Information files.

Funding: This study was supported by funding provided from the Japan Society for the Promotion of Science (No. 16H02708, 23KK0119, 24K03129). The funders had no role in study design, data collection and analysis, decision to publish, or preparation of the manuscript.

Competing interests: The authors declare no competing interests.

Abstract

In a national park in Northeast Thailand, agricultural land has been converted from natural forest by small-scale farmers for cassava agriculture. We hypothesise that long-termed cassava monoculture leads to soil degradation. To test the hypothesis, we conducted a five-year (2016-2020) study on the physical and chemical properties of soil in cassava farmland, and also examined the soil properties of its adjacent natural forests, as a control. Soil nitrogen, available phosphate and potassium, and bulk density were significantly higher in the farmland than in the forest, whereas cation exchange capacity, organic carbon, and clay fraction showed the opposite trend. The cation exchange capacity was positively correlated to the fraction of clay and the organic carbon within soil. These data indicate that the farmland soil was irreversibly degraded by the conversion from forest to cassava fields, through rapid decreases in cation exchange capacity, organic carbon, and the fraction of clay. For the farmland, soil nitrogen and bulk density increased significantly, whereas pH and organic carbon decreased significantly over the five years, indicating that these soil properties were gradually altered by repeated agricultural activities, such as fertilizer application and trampling. The use of glyphosate and paraquat herbicides is prohibited within national parks in Thailand. However, in 2020, glyphosate was detected in farmland soil (up to 5.0 mg kg^{-1}) during both the rainy and dry seasons, and glyphosate (2.5 mg l^{-1}) was detected in stream water from the farmland during the dry season. The use of glyphosate in the farmlands was thus suspected at least in 2020. Soil degradation and herbicide pollution may carry a high risk of causing irreversible changes in terrestrial ecosystems. We discuss the root causes of this issue from perspectives of agricultural production, economy, and the environmental impact, and propose effective policy measures.

Introduction

The global agricultural land area increased by 102 million hectare during the first two decades of the 21st century, corresponding to 9% of the agricultural land area in 2003[1]. During the 16 years from 2003 to 2019, the per capita agricultural land area decreased by 10% owing to population growth, while the per capita net primary production of agricultural land area increased by 3.5% owing to the enhancement and intensification of agriculture [1]. The increase in crop yields per capita and land area has resulted from the modernization of agriculture. Modernization relies on the development of agricultural facilities and cultivation techniques, development and utilization of agricultural machinery, introduction of improved crop varieties with higher yields, and long-term input of chemical fertilizers and pesticides in farmland. In particular, agricultural modernization is based on large-scale cultivation using a single-crop variety with a high yield (i.e., monoculture).

However, in homogeneous agriculture, diseases and pests are more likely to occur than in traditional agriculture [2]. In such monocultural farmlands, the heavy use of chemically synthesized pesticides and fertilizers is usually required for effective pest control and enhancement of crop yields [3]. In Thailand, the importation of herbicides increased by 10% over a four-year period, rising from 53615 tons in 2009 to 60232 tons in 2012, with glyphosate being the most imported herbicide, both in terms of cost and quantity [4]. Nevertheless, herbicides and pesticides have the potential to cause significant negative impacts on humans (including children), livestock, and the environment if mishandled [5-8]. Continuous attention is thus needed to evaluate and improve regulatory systems and their social implementation. However, in many developing countries, the effectiveness of regulatory systems is often low [6, 9]. Because of its broad effectiveness on both annual and perennial plants (including

various crops), glyphosate is popular in Thailand, particularly for effective weed control before planting, even in farmlands [4].

Cassava (*Manihot esculenta* Crantz) is well-suited to tropical and subtropical regions with warm temperatures [10]. This crop plant can grow even in nutrient-poor soil, drought, and heat environments [11] and has significant economic importance for local farmers in tropics [12-15]. Monocultural farming is a common practice worldwide for cassava cultivation. Such monocultural agroecosystems have various risks of soil degradation, including erosion on steep-slope farms, soil compaction and crusting, structural deterioration, flooding, organic carbon loss, microbial decline, salinization, and on-site and off-site damage [16]. Cassava roots penetrate only up to 0.5 m deep into the soil, but can extend 1-2 m horizontally depending on the planting density, contributing to the suppression of soil erosion [17]. However, some reports have showed that the loss of farmland soil is significant especially when cassava roots are harvesting [18-20]. On the effects of soil nutrients, cassava extracts significantly less nitrogen and phosphorus from the soil than most other crops while extracting similar amounts of potassium (K) [12]. Consequently, additional fertilization is needed to maintain soil fertility when cassava is continuously cultivated on the same land, especially K [13, 18, 21-23], phosphorus [18, 24, 25], and nitrogen [24], possibly leading to the overuse of fertilizers and an imbalance of nutrient levels within the farmland soil [26]. While once thought to be environmentally friendly, even low-input crops like cassava are now being recognized as contributors to cycles of environmental degradation that endanger future food production [15].

In terms of above-ground environmental factors, including air temperature, solar radiation, and precipitation in Northeast Thailand, the potential production of cassava can be further increased by adopting adequate water management, especially by

managing and expanding agricultural land in the mid-highlands [27]. However, we do not have sufficient information regarding soil factors or adequate management based on environmental conservation. In particular, long-term monitoring of soil quality in cassava farmlands is still rare. Here, we hypothesise that long-termed cassava cultivation leads to soil degradation. Additionally, we examined whether herbicides prohibited within Thailand's national parks were used. To sustainably obtain sufficient cassava yields, it is necessary to understand the extent of soil degradation caused by repeated cassava monocultures, and to know the actual impact of the long-term use of chemical fertilizers and pesticides, including herbicides, on soil and water. In the present study, we identify which soil properties incur irreversible damage due to agricultural activities, and furthermore, provide evidence to raise suspicions about the agricultural use of banned glyphosate.

Materials and Methods

Study site and method overview

This research was conducted in a cooperative research project of the Department of National Parks Wildlife and Plant Conservation, Thailand (Permission No. 5810701, 5810107, 5921601). All procedures in this study were carried out in accordance with the relevant guidelines of Valaya Alongkorn Rajabhat University. Phu Kao-Phu Phan Kham National Park (PKNP) is a national park located on the Khorat Plateau with sandy soil in Northeast Thailand. The study site (16°55'48.76"N, 102°27'40.12"E) is located in Phu Kao within this national park (Fig 1). Since 1998, the natural forests within this national park have been designated as protected areas for natural conservation. The Khorat Plateau is primarily composed of nutrient-poor sandy soil. On

this plateau, dry deciduous forests develop naturally [28, 29] and are composed of canopy trees that have adapted well to the oligotrophic sandy soils [30]. During the last nine years from 1991 to 2020, the yearly precipitation was 900-1300 mm (1252 mm on average), and the yearly mean air temperature was 21.1-32.5°C (26.0°C on average) at approximately 22.7 km southeast of the study site (based on the Thai Meteorological Department). There is a distinct dry season from November to January (Fig 1D).

In Phu Kao, there are three villages (Dong Bak, Wang Mon and Chai Mongkala) surrounded by agricultural lands, which are further enclosed by natural forests. Cassava monoculture has been predominantly carried out on agricultural land, which has been converted from natural forests since the 2000s. Although the boundary line between farmland has and protected natural forests was established in 2010 by the Thai government, agricultural land had expanded into protected forest areas beyond the boundary line during the successive five years (2011 to 2015) (Fig 1A). During the five years from 2016 to 2020, soil properties were examined at four locations (north, east, south, and west of the main village) in the cassava farmland. To further clarify the agricultural impact on soil, we examined the soil properties of the nearest forests at each of those four locations. The combination of comparisons between farmland and forest soils and long-term monitoring of farmland soil will clarify which soil properties are largely affected by agriculture.

Soil and water sampling

Soil samples were collected from at a depth of 15 cm from the soil surface, using a drop-hammer sampler equipped with double cylinders (ANS Inc., American Falls, ID, US). The size of inner cylinder was 4.5 cm in diameter and 15.2 cm in length (approximately 242 cm^3 in volume).

The farmland soil was collected after crop harvesting in the dry season, when the soil nutrient levels would be lowest in each growing season. Soil sampling was conducted at four locations (the north, east, south and west of the main village) (Fig 1A), and four subplots were subsequently set in each location (16 subplots in total). The positions of the 16 subplots were determined using a Global Positioning System (GPS; Garmin 60Csx, Garmin, Olathe, KS, USA), and the soil (0-15 cm thickness below the ground surface) was collected at the same positions three times (2016, 2018, and 2020). Furthermore, to compare the soil properties between the farmland and forest soils, we investigated the soil properties using soil samples collected from the nearest natural forests at the four locations during the dry season on November 15, 2020.

For nature conservation, the use of glyphosate and paraquat within national parks has been prohibited by the Thai government. During the dry season, stream water was blocked in the lower stream to obtain irrigation water. Because of the halted waterflow, pollutant accumulation was predicted during this season. To examine the possibility of herbicide pollution within farmland ecosystems, paraquat and glyphosate were tested in soil and stream water during the rainy (June 6) and dry seasons (November 18) in 2020. Farmland soils were collected from the 16 subplots. The water of the main stream flowing through the agricultural area was collected at three locations (upper, middle, and lower streams) with one sample taken at each location (Fig 1A).

Soil analysis

To examine the impact of long-term agricultural land use on farmland soil in Phu Koa, we examined 10 soil properties, soil texture (the fractions of sand, silt, and clay), cation exchange capacity, bulk density, pH, Kjeldahl nitrogen (N), available/exchangeable phosphorus (P), available/exchangeable potassium (K), organic carbon, electrical

conductivity (EC), and soil moisture contents, in monocultural cassava farmland over a five-year period (2016-2020). For soil physical properties, soil texture (dry mass %), bulk density (Mg m^{-3}), and soil moisture content (g g^{-3}) were examined.

To examine the soil texture, the collected soil was sieved with a 2-mm grid mesh, and subsequently air-dried. The volumetric composition of sand (> 20 μm in diameter), silt (2-20 μm) and clay (< 2 μm) in the soil samples were determined by the hydrometer method [31, 32] using an ASTM specific gravity hydrometer (Type 152H). The moisture contents were determined from air-dried soil samples as follows:

Soil moisture content = (soil wet mass – soil dry mass)/soil dry mass (1).

To examine the soil bulk density, the soil contained within the inner cylinder (242 cm^3) of the drop-hammer sampler was subsequently dried in an oven at 105°C for approximately 18-24 hours. The values of bulk density (Mg m^{-3}) were determined from the ratio of soil dry mass to the inner cylinder volume.

For chemical properties, the collected soils were air-dried, ground, and sifted through a 2-mm grid mesh before chemical analysis. The pH values in the soil solution extracted with distilled water were measured using a pH meter [33]. Soil N concentration (g kg^{-1}) was determined using the Kjeldahl method based on a wet combustion [34]. The available P concentration (mg kg^{-1}) was extracted with Bray II solution (0.03 N NH_4F/0.1 N HCl), and the concentration was colorimetrically examined with a spectrophotometer (U-2000, Hitachi Ltd., Tokyo, Japan) based on the reaction with ammonium molybdate and the development of molybdenum blue colour [35]. The available K concentration (mg kg^{-1}) was extracted using 1 molar NH_4OAc solution (pH 7.0), and the extracted K content was colorimetrically examined with a spectrophotometer (XP Flame Photometer, BWB Technologies Ltd, Newbury, UK) [36]. The soil organic carbon content (g kg^{-1}) was examined using the Walkley-Black chromic acid wet oxidation method, and the

oxidizable matter in the soil was oxidized using a 1 N potassium dichromate ($K_2Cr_2O_7$) solution [37]. Electrical conductivity (EC; mS m^{-1}) was measured with an EC meter (Soils Thermal Conductivity Meter, Model MK-TDS210-B, Hangzhou Asmik Sensors Technology Co., Ltd., Zhejiang, China) using a solution extracted with distilled water (1:5). The cation exchange capacity (cmol(+) kg^{-1}) was examined using the ammonium saturation method by saturating the soil with a solution of ammonium acetate (NH_4OAc, 1 M, pH 7) [38]. NH_4^+ replaces the cations that are absorbed by all of the clay micelles in the soil. The amount of NH_4^+ was determined by distillation after it was replaced by Na^+ from NaCl, which is a direct replacement method.

Assessing herbicide pollution

Water was collected from the main stream of the farmland. We added 0.5 ml of formic acid to 50 ml of the water samples and purified through the Osais HLB solid-phase extraction cartridge (Waters Corporation, MA, USA). The herbicide concentration in the water was examined as follows. We added 20 ml of 1% formic acid water/methanol to 20 g of soil sample (1:1 volume), and the mixture was shaken with a vortex mixer for 5 min. The samples were left to settle for 30 minutes and then centrifuged at 5,000 rpm for 5 min. A 5 ml sample of the supernatant was purified through the Osais HLB solid-phase extraction cartridge. To examine the paraquat and glyphosate concentrations, acetonitrile was used for one-step extraction and dewatering of the samples with $MgSO_4$ and purification with dispersive-solid-phase extraction (dispersive-SPE). Organic acids, other constituents, and excess water were removed using PSA (primary and secondary amine), C18-EC (end-capped) and $MgSO_4$, respectively [39]. Chemical analyses were conducted at 4°C for 24 h. The paraquat and glyphosate concentrations in

the soil and water were examined using high-strength liquid chromatography (UltiMate 3000 HPLC, Thermo Fisher Scientific, MA, US).

Statistics

For each soil property, significant differences between the agricultural and forest soils were examined using the pooled data from each site with one-way analysis of variance (ANOVA) (Table 1). The statistical variations among the five years (2016-2020) and among the four locations in each soil property were examined using repeated one-way ANOVA (Table 2). For soil properties with significant differences ($P < 0.05$) in Table 2, multiple comparisons using Tukey's honestly significant difference (HSD) test were conducted between the years and the locations (Table 3). To evaluate the contribution of the organic carbon, the fraction of clay, and pH to the cation exchange capacity, multiple correlation analysis was conducted, as follows: the cation exchange capacity was the dependent variable, and $\log_{10}$(organic carbon), $\log_{10}$(clay fraction), and pH were explanatory variables, employing a GLM assuming a Gaussian error distribution with an identity link function (Table 4).

All statistical analyses were conducted using R software [40] and RStudio (ver. 2023.06.1; RStudio, Boston, MA, USA). Statistical significance was recognized by $P < 0.05$ (***: $P < 0.001$, **: $P < 0.01$, *: $P < 0.05$, n.s.: $P \geq 0.05$).

Results

Soil properties

All raw data for the farmland and forest soils (0-15 cm depth) are represented in Supplementary Information, S1 and S2 Tables, respectively. The soil properties of farmland showed significant differences with those of adjacent forest soils in some

parameters (Fig 2, Table 1). For the soil texture, no significant differences were found in the fractions of sand and silt, whereas the fraction of clay was significantly lower in the farmland soil than in the forest soil. The sand fractions in the farmland and forest soils were 70.5% and 69.3%, respectively, indicating that the soil within this region was sandy because of the sandstone bedrock. Regarding soil chemical properties, Kjeldahl nitrogen (N; sum of organic N and ammonia N), available phosphorus (P), and available potassium (K) were significantly higher in the farmland soil than in the forest soil, whereas cation exchange capacity and organic carbon were significantly lower in the farmland soil. The average concentrations of N, P, and K in the farmland soil were 1.9 times, 3.1 times, and 6.3 times higher than those in the forest soil, respectively, and the cation exchange capacity of the farmland soil decreased by 71% compared with that of the forest soil. No significant differences were found in pH and EC between farmland and forest soils.

The annual variations in soil properties in the farmland over the five years (2016-2020) are shown in Fig 3 (see Tables 2, 3 for statistics). No significant yearly variations were found in available P, available K, cation exchange capacity, and the clay fraction. Soil Kjeldahl N and bulk density in 2018 and/or and 2020 increased significantly from those in 2016, whereas soil organic carbon, pH, and moisture content decreased significantly. Among the locations, no significant variations were not found for all soil properties, except for the organic contents between the south (S) and north (N) sites (Table 3, S1 Fig).

Both cation exchange capacity and the fraction of clay were significantly lower in the farmland than in the forest (Fig 2), and no yearly variations were found in these soil properties (Table 2). Assuming that the soil before deforestation was similar to the examined forest soil, the current data showed that the conversion to cassava fields

resulted in a rapid decrease in cation exchange capacity and clay fraction within several years before starting this observation (at least the period from 2011 to 2016), followed by a delayed decrease in organic carbon (Fig 3D). The significant decrease in pH was found in only 2020 (last year) (Fig 3E). Over all, the cation exchange capacity was significantly and positively correlated to $\log_{10}$(organic carbon), $\log_{10}$(clay fraction), and pH (Fig 4). The results of the multiple correlation analysis indicated that the contribution to cation exchange capacity increased in the following order: pH, the fraction of clay, and organic carbon (Table 4).

Paraquat and glyphosate pollution

In 2020, we assessed glyphosate and paraquat in farmland soil and the water of the main stream in farmland (Table 5). Paraquat was undetectable in both the soil and stream water. Unfortunately, glyphosate in the farmland soil was detected at levels of up to 5.0 mg kg^{-1} in both the rainy and dry seasons throughout at all locations. In the main stream, glyphosate (2.5 mg l^{-1}) was observed in the water of the middle stream in the dry season, when waterflow had stopped. In the rainy season, glyphosate was not detected in the stream water, probably because of continuous waterflow. It was confirmed that glyphosate was widely used within farmlands, at least in 2020.

Discussion

Soil degradation

Significant differences in soil health were found between the forest and the cassava farmland soils. The cation exchange capacity is a measure of the ability to adsorb and retain cations in soil, showing that as the values increase, the soil can retain more nutrient ions. Both the cation exchange capacity and the clay fraction of forest soil was

1.4 times higher than that of farmland soil (Fig 2), and no significant variations were found across years for the farmland soil (Table 2). Soil organic of forest soil was 2.5 times higher than that of farmland soil, and the soil organic contents of farmland decreased yearly (Fig 2). The conversion from forest to cassava fields resulted in rapid decreases in cation exchange capacity and clay fraction, followed by delayed decreases in organic carbon and pH. Usually, the cation exchange capacity is affected by clay fraction [41 Ohta 2000] and organic contents and pH [42 Haynes 1998, 43 Jarecki 2005]. The cation exchange capacity significantly decreased with decreasing the fraction of clay, the organic carbon, and pH (Fig 4). These facts indicate that the conversion from natural forests to farmland results in rapid and irreversible soil degradation through a decline in cation exchange capacity, associated with decreases in clay fraction, organic carbon and pH.

Soil nutrients

Cassava can grow even in nutrient-poor soil, but to obtain sufficient yields, it is necessary to apply fertilizers containing many elements including K, N, and P [13, 18, 21-25]. In Thailand, non-fertilizer treatment over a period of 15-30 years induces a decrease of 30-40% from the initial cassava yield per unit area [12]. For the yearly change of farmland soil properties, Kjeldal N and bulk density increased significantly, whereas pH, organic carbon, and moisture content decreased significantly (Fig 3). These yearly changes would be due to repeated agricultural activities, and farmers have been continuously applying excess N fertilizer to maintain sufficient cassava yields. The increase in bulk density indicates soil compaction caused by repeated agricultural trampling, possibly reducing cassava growth through inhibition of root penetration and nutrient uptake [44, 45]. The average concentrations of Kjeldal N, available P, and

available K in the farmland soil were 1.9 times, 3.1 times and 6.3 times higher than those in the forest soil, respectively (Fig 2). The increase in soil N and acidification could be due to the overuse of chemical fertilizers, compensating for nutrient loss throughout the repeated crop harvesting with artificial fertilization. It has known that additional K fertilization leads the increases in the number and diameter of cassava roots [23], and soil P and N are the major limiting factors of plant growth, particularly in sandy oligotrophic soil of Thailand [30].

Excess chemical N fertilizer leads to soil acidification, which is a major global issue of sustainable development for agricultural ecosystems [46, 47]. Although no significant variations were not found in pH values between the farmland and forest soils (Table 1), the pH values of 2020 (5.4 in average) were significantly lower than those of 2016 (6.0 in average) and 2018 (5.8 in average) for the farmland soil (Fig 3). Aluminum (Al) is the third most abundant element in the Earth's crust, following oxygen and silicon, and it is universally present in soils [48]. Al in soil begins to leach out at pH below 5.5 and then Al^{3+} ions become the predominant form at pH below 4.5, inducing the inhibition of root elongation in plants [49]. Cassava is less sensitive to acid soils than grain crops, such as maize (*Zea mays* L.), on Oxisoles, Ultisols, and Alfisols of tropics [50]. However, the prolonged exposure of cassava roots to high Al^{3+} levels gradually and continuously promotes proton release from their roots and leads to the dissolution of Al^{3+} ions and soil acidification, resulting in high metal toxicity and low P availability [47]. Therefore, it is recommended to keep the soil pH below 5.5 even in cassava, to mitigate aluminum dissolution and the toxicity caused by high Al^{3+} ions [47].

In addition, the overuse of chemical fertilizers rapidly reduces soil organic carbon due to soil acidification and microbial decline [51]. At the present study site, the

organic carbon of forest soil was 2.5 times higher than that of farmland soil on average (Fig 2). Soil organic carbon plays a crucial role in governing not only cation exchange capacity [42, 43] but also the physicochemical reactions that regulate the availability of micronutrients (such as Zn, Cu, Fe, Mn, B, and Mo) [52]. The cation exchange capacity is usually defined as the amount of positive charge that can be exchanged per soil mass. The values (mean ± 1 S.D.) of cation exchange capacity in the forest and farmland soils were 8.0 ± 3.4 and 5.0 ± 2.2 cmol (+) kg^{-1}, respectively (Fig 1). Not only these values of cation exchange capacity but also those of N and P concentrations at this study site are extremely low and comparable to the values of degraded or sandy soil in East Kalimantan, Indonesia [41], indicating that the Khorat Plateau (the present study site) originally had oligotrophic soil because of the sandy soil [30].

Such oligotrophic soil and soil degradation advanced by the cassava monoculture resulted in low crop yields, consequently increasing the need for chemical fertilization. Tropical soils generally lack resilience once degraded [53]. Soil degradation and chemical pollution thus pose a high risk of making ecosystems irreversibly unable to return to their original state. The nitrogen remaining in the soil undergoes transformation into nitrate ions (NO_3^-) within the soil, and NO_3^- readily dissolves in water. Nitrogen loading in the nutrient-poor sandy soil of natural forests may have unexpected effects on canopy trees that have adapted to oligotrophic soil for a long time [30]. The soil degradation caused by repeated cassava monocultures can thus be a major driver of negative feedback in local-scale environmental conservation. Furthermore, dissolved NO_3^- facilitates leaching from the soil into groundwater and river water. Eutrophication can occur when a significant influx of nutrients occurs in rivers, lakes, and seas.

Herbicide use

The use of pesticides has been increased worldwide, thereby increasing the risk of environmental exposure. Although the direct toxicity of glyphosate to humans is low, the risk of inducing cancer remains controversial and several glyphosate hotspots in the soil have been detected across the European Union [54]. In Thailand, almost all insecticides and herbicides are imported [6]. Since June 1, 2020, the use of chlorpyrifos and paraquat was prohibited, whereas the application of glyphosate was permitted under specific restrictions governed by the requirement of the maximum residue level (MRL). Since June 30, 1998, the use of all herbicides has been prohibited in all forest reserves and their watershed areas in Thailand, to prevent hazards and contamination in natural water sources due to spread by wind and water erosion [55-57]. Several recent experiments exposing small mammals (rats) to glyphosate have shown that even low concentrations of glyphosate can cause significant damage to offspring in successive generations (F2 and F3) rather than the parental generation (F1) because of abnormal DNA methylation of the germline (e.g., sperm) [58-60].

Although the half-life of glyphosate in soil ranges between 2 and 197 days, the illegal use of glyphosate in this cassava farmland was recognized even within the national park at least in 2020 (Table 5). To sustain the increase in family members, the use of banned herbicides would provide an easy and cost-effective solution for local farmers to increase cassava yields. Nevertheless, investment in chemical fertilizers accounts for 53% of their production costs [28]. Herbicides, such as glyphosate, are used worldwide to control weeds. Although Thailand has not yet approved genetically modified crops (GOC) that can tolerate glyphosate [4], glyphosate-resistant weeds have been rapidly expanding worldwide owing to the excessive use of GOC and glyphosate [61, 62]. The development of alternative herbicides has also been attempted [63, 64],

and low-cost generic pesticides and herbicides are readily available worldwide. However, a decrease in pesticide costs may result in an increase in the frequency of its application. Furthermore, because of the quality variations among generic pesticides depending on the manufacturing process and raw materials, it is crucial to ensure their effectiveness and safety through regulation and management on a global scale [9].

Socioecological perspectives

This case study from Northeast Thailand presented here is a common issue faced by developing countries worldwide generally face. To address this problem and achieve sustainable socioecosystems, it is necessary to comprehensively consider the root causes of this issue, including population growth in tropical regions, agricultural production, and the economy, and their impacts on the environment.

Over the last decade, the growth of the human population has resulted in the remarkable expansion of cassava farmland by local-scale farmers, beyond the boundary line between farmland and protected natural forests that set by the Thai government in 2010 (see Fig 1) [37]. Significant tree species loss was also found within the protected natural forest beside the farmland [28]. The processes of urbanization and agricultural development have had a significant impact on not only the natural environment but also on local socioeconomic factors, including the labour force, education expenses, employment opportunities, household commodities, and vehicles [65-67]. In Thailand, the economic growth rate reached approximately 7.8% per year during the period of the Seventh National Economic and Social Development Plan (1992-1996), whereas the annual growth rate in the country's agricultural sector during the same period was only 2.5% [67]. Certainly, the rate of poverty reduction is closely related to the economic growth rate while facing a consistent increase in income inequality [68]. The

proportions of farmland area and forests are 40.7% and 25.3%, respectively, of the country's area (based on the Ministry of Agriculture and Cooperation), and 44.6% of the workforce is involved in the agricultural sector, but contributes only 7.2% of the country's GDP (National Economic and Social Development Board data). Although approximately 67% of the human population resides in rural areas in Thailand (1995-2000), many agricultural lands have been converted into industrial zones, luxury resorts, and towns, resulting in an overall shortage of agricultural land [67]. Nevertheless, a large proportion of the population in Thailand is still engaged in agriculture, which serves as a significant source of income.

To ensure food security and production, the development of new agroecosystems based on environmental conservation is required in the tropics overall. A transition from monocultural cassava cultivation to new farmland use, including the idea of traditional agricultural systems, is necessary. Although traditional slash-and-burn farming has only low productivity, additional chemical fertilizers and herbicides are not needed, having the advantages of low cost and low soil degradation risks [53, 69]. A recent meta-analysis showed that crop diseases decrease and crop yields increase with increasing diversity in intraspecific cultivar mixtures [2]. Another meta-analysis of cassava-based agroecosystems indicated that interspecies cropping had provides positive effects on several key ecosystem services, including pest suppression, disease control, and soil- and water-related services [16]. Experimentally, mixed cropping systems, including intercropping and crop rotation systems, have been shown to play a significant role in increasing cassava yields [17, 70-72]. Thus, diversified agriculture can thus play a significant role in ensuring a balance between productivity, crop resilience, and environmental health [73, 74]. Furthermore, the use of organic mulch as an alternative to plastic mulch and chemical herbicides is effective for maintaining crop

yields through adequate or moderate weed control [75] and soil improvement effects [42], potentially reducing the use of chemical fertilizers and herbicides [76, 77].

Nevertheless, in social implementation, political systems that support income and promote diverse crop cultivation are important, particularly for small-scale farmers. Enhancing ecological agricultural systems, using multiple crops with various adaptive characteristics will be effective not only for maintaining human health and well-being but also for strengthening resilience to climate change, thereby improving food security in developing countries [15, 73]. Adopting agricultural systems that promote ecosystem services enhances resistance not only to pest and disease control but also to climate change, reducing the risk of decreased crop production due to climate change and contributing to the development of a resilient society.

Conclusion

The comparison of farmland and forest soils, coupled with long-term monitoring of farmland soil, effectively described the characteristics of soil degradation resulting from repeated cassava monoculture. The significant decreases in clay fraction, organic carbon, and cation exchange capacity in the farmland soil indicate that the conversion from forest to farmland leads to rapid and irreversible soil degradation. For the yearly change in the farmland, soil N and bulk density were increasing, whereas organic carbon and pH were decreasing significantly. Although these soil properties may be relatively quickly recovered by cessation of soil fertilization and repeated agricultural trampling, these agricultural activities will be required to maintain sufficient cassava yields. Because the farmland is located within a national park, the use of herbicides is prohibited. However, we provide evidence of the illicit use of herbicides and the unauthorized expansion of agricultural activities into protected natural forests. For

nature conservation, it is necessary to establish political systems that support local farmers financially, promote the transition to diverse crop cultivation, and encourage the use of organic mulch.

Supporting information

S1 Table. Raw data for the farmland soil properties. (XLSX)

S2 Table. Raw data for the forest soil properties. (XLSX)

S1 Fig. Variations in soil properties among the locations for the farmland. (PDF)

Acknowledgements

We wish to thank Mr. Y. Kanzaki for drawing Figure 1. This study was a cooperative research project of the Department of National Parks Wildlife and Plant Conservation.

Author Contributions

Conceptualization: Ananya Popradit, Marcel Robischon, Arichai Wanasiri

Formal analysis: Shin-Taro Saiki, Ananya Popradit

Project administration: Ananya Popradit, Arichai Wanasiri

Investigation: Ananya Popradit, Yutthana Nakhokwik

Writing – original draft: Atsushi Ishida, Ananya Popradit, Jin Yoshimura

References

1. Potapov P, Turubanova S, Hansen MC, Tyukavina A, Zalles V, Khan A, et al. Global maps of cropland extent and change show accelerated cropland expansion in the twenty-first century. Nature Food. 2022; 3: 19-28. doi.org/10.1038/s43016-021-00429-z

2. Reiss ER, Drinkwater LE. Cultivar mixtures: a meta-analysis of the effects of intraspecific diversity on crop yield. Ecol Appl. 2018; 28: 62-77. doi.org/10.1002/eap.16292018

3. Lithourgidis AS, Dordas CA, Damalas CA, Vlachostergios DN. Annual intercrops: an alternative pathway for sustainable agriculture. Aust J Crop Sci. 2011; 5: 396-410.

4. Suvittawat A. Gyphosate market in Thailand: opportunities and challenges. Inf Manag Bus Rev. 2014; 4: 207-211. doi.org/10.22610/imbr.v6i4.1116

5. Bai SH, Ogbourne SM. Gyphosate: environmental contamination, toxicity and potential risks to human health via food contamination. Environ Sci Pollut Res. 2016; 3: 18988-19001. doi.org/10.1007/s11356-016-7425-3

6. Laohaudomchok W, Nankongnab N, Siriruttanapruk S, Klaimala P, Lianchamroon W, Ousap P, et al. Pesticide use in Thailand: Current situation, health risks, and gaps in research and policy. Hum Ecol Risk Asses. 2020; 27: 1147-1169. doi.org/10.1080/10807039.2020.1808777

7. Berkal MA, Nardinm C. Pesticide biosensors: trends and progresses. Anal Bioanal Chem. 2023; 415: 5899-5924. doi.org/10.1007/s00216-023-04911-4

8. Grammenou A, Petropoulos SA, Thalassinos G, Rinklebe J, Shaheen SM, Antoniadis V. Biostimulants in the soil-plant intertace: Agro-environmental implications - A review. Earth System Environ. 2023; 7: 583-600. doi.org/10.1007/s41748-023-00349-x

9. Haggblade S, Diarra A, Traoré A. Regulating agricultural intensification: Lessons from west Africa's rapidly growing pesticide markets. Dev Policy Rev. 2022; 40: e12545. doi.org/10.1111/dpr.12545

10. El-Sharkawy MA. Global warming: causes and impacts on agro-ecosystems productivity and food security with emphasis on cassava comparative advantage in the tropics/subtropics. Photosynthetica. 2014; 52: 161-178. doi.org/10.1007/s11099-014-0028-7

11. El-Sharkawy MA. Cassava biology and physiology. Plant Mol Biol. 2004; 56: 481-501. doi.org/10.1007/s11103-005-2270-7

12. Howeler RH. Long-term effect of cassava cultivation on soil productivity. Field Crops Res. 1991; 26: 1-18. doi.org/10.1016/0378-4290(91)90053-X

13. Howeler RH. Developing systainable cassava production systems with farmaers' involvement in Asia. In: Fujisaka S, editor. Systems and Farmaer Participatory Research. Developments in Research on Natural Resource Management. CIAT Publication; 1999. pp. 86-102.

14. Choenkwan S, Fox JM, Rambo AT. Agriculture in the mountains of northeastern Thailand: currebt situation and prospects for development. Mt Res Devl. 2014; 34: 95-106. doi.org/10.1659/MRD-JOURNAL-D-13-00121.1

15. Reynolds, TW, Waddington SR, Anderson CL, Chew A, True Z, Cullen A. Environmental impacts and constraints associated with the production of major food crops in Sub-Saharan Africa and South Asia. Food Security. 2015; 7:795-822. doi.org/10.1007/s12571-015-0478-1

16. Delaquis E, de Haan S, Wyckhuys KAG. On-farm diversity offsets environmental pressures in tropical agro-ecosystems: a syntehtic review for cassava-based systems. Agric Ecosyst Environ. 2018; 251: 226-235. doi.org/10.1016/j.agee.2017.09.037

17. Iijima M, Izumi Y, Yuliadi E, Sunyoto S, Ardjasa WS. Cassa-based intercropping systems on Sumatra island in Indonesia: productivity, soil erosoion, and rooting zoon. Plant Prod Sci. 2004; 7: 347-355. doi.org/10.1626/pps.7.347

18. Oshunsanya O, Sammson VW, Yu H. Soil loss due to harvesting of peanut and cassava under traditional farming systems: Cost implicatios of soil nutrient loss. J Soil Water Conserv. 2022; 77: 240-248. doi.org/10.2489/jswc.2022.00111

19. Sumithra R, Thushyanthy M., Srivaratharasan T. Assesment of soil loss and nutrient depletion due to cassava harvesting: A case study from inout traditional agriculture. Int Soil Water Coserv Res. 2013; 1: 72-79. doi.org/10.1016/S2095-6339(15)30041-1

20. Kuhwald M, Busche F, Saggau P, Duttmann R. Is soil loss due to crop harvesting the most disregarded soil erosion process? A review of harvest erosion. Soil Tillage Res. 2022; 215: 105213. doi.org/10.1016/j.still.2021.105213

21. Howeler RH, Cadavid LF. Short- and long-term fertility traits in Colombia to determine the nutrient requirements of cassava. Nutr Cycl Agroecosystems. 1990; 26: 61-80. doi.org/10.1007/BF01048744

22. Chua MF, Youbee L, Oudthachit S, Khanthavong P, Veneklaas EJ, Malik AI. Pottassium fertilisation is required to sustain cassava yield and soil ferttility. Agronomy. 2020; 10: 1103. doi.org/10.3390/agronomy10081103

23. Gonçalves YS, Freitas MSM, de Carvalho AJC, Vieira ME, Peçanha DA, Cunha JM, et al. Potassium sources impact on cassava plant productivity, quality and mineral composition. J. Plant Nutr. 2022; 45: 86-94. doi.org/10.1080/01904167.2021.1949465

24. Putthacharoen S, Howeler RH, Jantawat S, Vichukit V. Nutrient uptake and soil erosion losses in cassava and six other crops in a Psamment in Eastern Thailand. Field Crops Res. 1998; 57: 113-126. doi.org/10.1016/S0378-4290(97)00119-6

25. Leitchm A, Anusontpornperm A, Thanachit S, Jindaluang W, Phun-Iam M. Cassava response to phosphorus fertilizer in Warin soil series amended with cassava tails and stalk-bontonite mixture. Trends Sci. 2023; 20: 4885. doi.org/10.48048/tis.2023.4885

26. Setyastika, US, Utami SR, Kurniawan S, Agustina C. Is long-term cassava-based agriculture sustainable? (Case study of potassium content in the soil). IOP Conf Ser: Earth Environ Sci. 2023; 1131: 012003. doi.org/10.1088/1755-1315/1131/1/012003

27. Nawata E, Nagata Y, Kono Y. Iwama K, Yamamoto T, Watanabe K, et al. Mapping of cassava productivity using GIS in Northeast Thailand. Jpn J Trop Agr. 2004; 48: 211-219. doi.org/10.11248/jsta1957.48.211

28. Phromma I, Pagdee A, Popradit A, Ishida A, Uttaranakorn S. Protected area co-management and land use conflicts adjacent to Phu Kao–Phu Phan Kham National Park, Thailand. J Sustain For. 2019; 38: 486-507. doi.org/10.1080/10549811.2019.1573689

29. Popradit A, Srisatit T, Kiratiprayoon S, Yohsimura J, Ishida A, Shiyomi M, et al. Anthropogenic effects on a tropical forest according to the distance from human settlements. Sci Rep. 2015; 5: 14689. doi.org/10.1038/srep14689

30. Ishida A, Yamaji K, Nakano T, Ladpala P, Popradit A, Yoshimura K, et al. Comparative physiology of canopy tree leaves in evergreen and deciduous forests in Thailand. Sci Data. 2023; 10: 601. doi.org/10.1038/s41597-023-02468-6

31. Burt, R. Soil Survey Laboratory Methods Manual. Soil Survey Labratory Investigations Report No. 42. USDA-NRCS; 2004.

32. Jensen JL, Schjønning P, Watts CW, Christensen BT, Munkholm LJ. Soil texture analysis revisited: Removal of organic matter matters more than ever. PLoS ONE. 2017; 12; e0178039. doi.org/10.1371/journal.pone.0178039

33. Soil Survey Staff. Keys to Soil Taxonomy. 12th Edition. USDA-NRCS; 2014.

34. Bremmer JM, Mulvaney CS. Total nitrogen. In: Page AL, Miller RH, Keeny DR, editors. Methods of Soil Analysis. Society of Agronomy and Soil Science Society of America; 1982. pp. 1119-1123.

35. Murphy J, Riley JP. A modified single solution method for the determination of phosphate in natural waters. Anal Chim.Acta. 1962; 27: 31-36. doi.org/10.1016/S0003-2670(00)88444-5

36. Jackson MLR, Barak P. Soil Chemical Analysis: Advanced Course. University of Wisconsin-Madison Libraries Papallel Press; 2005.

37. Mylavarapu R. Walkey-Black. Unit 5 Soil Organis Matter. In: Sikora FJ, Moore KP, editors. Soil Test Methods from the Southeastern United State. Southern Cooperative Series Bulletin No. 419. The Southern Association of Agricultural Experiment Station Directors; 2014. pp. 158-161. https://aesl.ces.uga.edu/Sera6/PUB/Methodsmanualfinalsera6.Pdf

38. Landon JR. Booker tropical soil manual: A handbook for soil survey and agricultural land evaluation in the tropics and subtropics. London, Routledge; 1991. https://doi.org/10.4324/9781315846842

39. Anastassiades M, Lehotay SJ, Štajnbaher D, Schenck FJ. Fast and easy multiresidue method employing acetonitrile extraction/partitioning and "Dispersive solid-phase extraction" for the determination of pesticide residues in produce. J AOAC Int. 2003; 86: 412-131. doi.org/10.1093/jaoac/86.2.412

40. R Development Core Team. 2019 R: A language and environmental for statistical computing. R Foundation for Statistical Computing. Available from https://https://www.R-project.org/

41. Ohta S, Morisada K, Tanaka N, Kiyono Y, Effendi S. Are soils in degradaded dipterocarp forest ecosystems deteriorated? A comparison of Imperata grasslands, degraded secondary forests, and primary forests. In: Guhardja E, Fatawi M, Sutisna M, Mori T, Ohta S, editors. Rainforest Ecosystems of East Kalimantan. Tokyo, Springer; 2000. pp 49-57.

42. Haynes RJ, Naidu R. Influence of lime, fertilizer and manure applications on soil organic matter content and soil physical conditions: a review. Nutr Cycl Agroecosyst. 1998; 51: 123-137. https://doi.org/10.1023/A:1009738307837

43. Jarecki MK, Lal Rattan, James R. Crop management effects on soil carbon sequestration on selected farms' fields in northeastern Ohio. Soil Tillage Res. 2005; 81: 265-276. https://doi.org/10.1016/j.still.2004.09.013

44. Kolawole OK, Awodum MA, Ojeniyi SO. Soil fertility improvement by *Tithonia diversifolia* (Hemsl.) A Gray and its effect on cassava performance and yield. Int J Eng Sci. 2014; 3: 36-43.

45. Bilong EG, Abossolo-Angue M, Ajebesone FN, Anaba BD, Madong BA, Nomo LB, et al. Improving spoil physical properties and cassava productivity through

organic management in the southern Cameroon. Neliyon. 2022; 8: e09570. doi.org/10.1016/j.heliyon.2022.e09570

46. Fageria NK , Nascente AS. Management of soil acidity of South American soils for suitable crop production. Adv Agronomy. 2014; 128:221-275. doi.org/10.1016/B978-0-12-802139-2.00006-8

47. Punpom T, Leksungnoen P, Aramrak S, Kongsil P, Wisawapipat W. Triggering root proton efflux as an aluminum-detoxifying mechanism in cassava. Ann Agric Sci. 2022； 67: 173-180. https://doi.org/10.1016/j.aoas.2022.10.002

48. von Uexküll HR, Mutert E. Global extent, development and economic impact of acid soils. Plant Soil. 1995; 174: 1-15. doi.org/10.1007/BF00009558

49. Horst WJ , Wang Y, Etica D. The role of the root apoplast in aluminium-induced inhibition and in aluminium resistance of plants: a review. Ann Bot. 2010; 106: 185-197. doi.org/10.1093/aob/mcq053

50. Aune, JB, Lal R. Agricultural productivity in the tropics and critical limits of properties of Oxisols, Ultisols, and Alfisols. Trop Agric 1997; 74: 96-103. https://journals.sta.uwi.edu/ojs/index.php/ta/article/view/2488

51. Food and Agriculture Organization for the United Nations (FAO). Soil for nutrition: state of the art. Rome, FAO; 2022. https://https//www.fao.org/3/cc0900en/cc0900en.pdf

52. Dhaliwal SS, Narech RK, Mandal A, Singh R, Dhaliwai MK. Dynamics and transformations of micronutrients in agricultural soils as influenced by organic matter build-up: A review. Enviorn Sustaina Indic. 2019; 1-2; 100007. doi.org/10.1016/j.indic.2019.100007

53. Are KS, Oluwatasin GA, Adeyolanu OD, Oke AO. Slash and burn effect on soil quality of Alfispl : Soil physical properties. 2009; Soil Tillage Res. 103: 4-10. doi.org/10.1016/j.still.2008.08.011

54. Silva V, Montanarella L, Jones A, Fernández-Ugalde, Mol HGJ, Ritsema CJ, et al. Distributionof glyphosate and aminomethylphosphnic acid (AMPA) in agricultural topsoils of the European Union. 2018; Sci Total Environ. 62; 1352-1359. doi.org/10.1016/j.scitotenv.2017.10.093

55. Royal Gazette. Announcement of the Ministry of Agriculture and Cooperatives regarding discontinuing the use of labels and containers for hazardous substances related to glyphosate under the responsibility of the Department of Agriculture. 2019. (in Thai language) Available form: https://www.opsmoac.go.th/buengan-dwl-files-412891791918

56. Royal Gazette. Ministry of Industry Announcement Regarding the list of hazardous substances (6th edition). 2020. (in Thai language) Available form: https://www.ratchakitcha.soc.go.th/DATA/PDF/2563/E/117/T_0056.PDF

57. Royal Gazette. Regulations of the Department of National Parks, Wildlife and Plant Conservation on concerning on access to national parks. 2021. (in Thai language) Available form: https://portal.dnp.go.th/DNP/FileSystem/download?uuid=513724c5-98d9-4f18-a347-2799d1e20cea.pdf

58. Milesi MM, Lorenz V, Pacini G, Repetti, Demonte LD, Varayoud J, et al. Perinatal exposure to a glyphosate-based herbicide impaires female reproductive outcomes and induces second-generation adverse effects in Wistar rats. Arch. Toxicol. 2018; 93: 3635-3638. doi.org/10.1007/s00204-019-02609-0

59. Lorenz V, Milesi MM, Schimpf MG, Luque EH, Varayoud J. Epigenetic disruption of estrogen receptor alpha is induced by a glyphosate-based herbicide in the preimplantation uterus of rats. Mol Cell Endocrinol. 2019; 15: 133-141. doi.org/10.1016/j.mce.2018.120.022

60. Kusbad D, Nilsson EE, King SE, Sadler-Riggleman I, Beck D, Skinner MK. Assessment of glyphosare induced epigenetic transgenerational inheritance of pathlogies and sperm epimutations: generational toxicologt. Sci Rep 2019; 9: 6372. doi.org/10.1038/s41598-019-42860-0

61. Heap I. Global perspective of herbivide-resistant weeds. Pest Manag Sci. 2014; 70: 1306-1315. doi.org/10.1002/ps.3696

62. Squires CC, Coleman GRY, Broster JC, Preston C, Boutsalis P, Owen MJ, et al. Increasing the value and effciency of hebicide resistance surveys. Pest Manag Sci 2021; 77: 3881- 3889. doi.org/10.1002/ps.6333

63. Tahmasebi BK, Alebrahim MT, Roldán-Gómez RA, da Silveira HM, de Carvalho LB, la Cruz RA, et al. Effectiveness of alternative herbicides on three Conyza species from Europe with and without glyphosate resistance. Crop Prot. 2018; 112: 350–355. doi.org/10.1016/j.cropro.2018.06.021

64. Kanatas P, Anthonopoulos N, Gazoulis I, Travlos IS. Screening glyphosate alternative weed control options in important perennial crops. Weed Sci. 2021; 69: 704–718. doi.org/10.1017/wsc.2021.55

65. Garip F. The impact of migration and remittances on wealth accumulation and distribution in rural Thailand. Demograpy 2014; 51; 673-698. doi.org/10.1007/s13524-013-0260-y

66. Gutzler C, Helming K, Balla D, Dannowski R, Deumlich D. Glemnitz M, et al. Agricultural land use changes–a scenario-based sustainability impact assessment for Brandenburg, Germany. Ecol Indic. 2015; 48; 505-517. doi.org/10.1016/j.ecolind.2014.09.004

67. Chingprapa A, Maneeratana T, Dirdjosubroto W. Situation, needs and interests of rural young people: The case of Thailand. In: Report of the Inter-Country Worksjop on Population Education for the Rural Youth. Rome, DOAE-FAO; 1997. https://https://www.fao.org/3/X5630e/X5630e04.htm

68. Warr P. Poverty reduction through long-term growth: the Thai experience. Asian Econ Papers 2009; 8: 51-76. doi.org/10.1162/asep.2009.8.2.51

69. Kukla J, Whitfeld T, Cajthaml T, Baldrian P, Veselá-Šimáckovâ H, Novotný V, et al. The effect of traditional slash-and-burn agriculture on soil organic matter, nutrient content, and microbiota in tropical ecosystems of Papua New Guinea. Land Degrad Dev 2019; 30: 66-177. doi.org/10.1002/ldr.3203

70. Lal R, Ghuman BS, Shearer W. Cropping systems effects of a newly-cleared untisol in Southern Nigeria. Soi Technol. 1992; 5: 27-38. doi.org/10.1016/0933-3630(92)90004-K

71. Ardjasa WS, Ando H, Kimura M. Yield and soil erosion among sassava-baswed cropping patterns in South Sumatra. Soil Sci Plant Nutr. 2001; 47: 101-112. doi.org/10.1080/00380768.2001.10408372

72. Kuyper TW. Intercropping and crop rotations in cassava cultivation: a production systems approch. Cambride, Burleigh Dodds Cience Publishing Ltd; 2017.

73. Brenda BL. Resilience in agriclture through crop diversiication: Adaptive management for environmental change. BioScience 2011; 61: 183-193. doi.org/10.1525/bio.2011.61.3.4

74. Zabel F, Müller, Elliott J, Minoli S, Jägermeyr J, Schneider JM, et al. Large potential for crop production adaptation depends on available future varieties. Glob Chang Biol. 2021; 27: 3870-3882. doi.org/10.1111/gcb.15649

75. Horimoto S, Fukuda K, Yoshimura J, Ishida A. Fresh-marketable tomato yields enhanced by moderate weed control and supressed fruit dehiscence with woodchip mulching. Sci Rep. 2022; 12: 13283. doi.org/10.1038/s41598-022-15568-x

76. Hartwing NL, Ammon HU. Cover crops and living mulches. Weed Sci. 2002; 50: 688-699. doi.org/10.1614/0043-1745(2002)050[0688:AIACCA]2.0.CO;2

77. Samedani B, Ranjbar M, Rahimian H, Jahansoz MR. Utilization of rye and hairy vetch cover crops for weed control in transplanted tomato. Pak J Biol Sci. 2006; 9: 2323-2327. doi.org/10.3923/pjbs.2006.2323.2327

Figure legends

Fig 1. A map of Phu Koa, the photos of monocultural cassava farmland and the seasonal variations of precipitation and air temperature. (**A**) The maps of Phu Kao and Thailand. Green areas show natural forest and light-yellow areas show crop farmland (mainly by cassava) including human villages, and the light-blue lines show streams. The red lines indicate the boundary set by the Department of National Parks, Wildlife and Plant Conservation (DNP) in 2010, to legally separate the farmland and protected forest areas. Four red-points indicate the sampling sites of farmland and forest soil (north, east, south, and west of the main village), and three blue-points indicate those of stream water (upper, middle, and lower streams). The photos are the cassava farmland (**B**) before harvesting in the rainy season and (**C**) after harvesting in the dry season (by A. Popradit). (**D**) The monthly precipitation (bars) and the monthly averaged air temperature (closed cycles) during the research period (2016 to 2020). Vertical vars show ± 1 S.D.

Fig 2. The soil properties of the farmland and its adjacent forest soils. (**A**) Kjeldahl nitrogen (the sum of organic N and ammonia N; g kg^{-1}), (**B**) available phosphorus (P; mg kg^{-1}), (**C**) available potassium (K; mg kg^{-1}), (**D**) organic carbon (g kg^{-1}), (**E**) pH, (**F**) electrical conductivity (EC; mS m^{-1}), (**G**) cation exchange capacity (cmol(+) kg^{-1}), (**H**) fraction of sand (%), (**I**) fraction of silt (%), (**J**) fraction of clay (%), (**K**) bulk density (Mg m^{-3}), and (**L**) moisture content (g g^{-1}). The boxplot shows the median (horizontal bar), interquartile range (box), 5th and 95th percentiles (whiskers; vertical bars), and outliers (black closed points). Different letters (a, b, c) show the significant differences among the years, as follows: ***: $P < 0.001$, **: $P < 0.01$, *: $P < 0.05$, and n.s.: $P \geq 0.05$.

Fig 3. The yearly change in soil properties after crop harvesting in the dry season. (**A**) Kjeldahl nitrogen (the sum of organic N and ammonia N; g kg^{-1}), (**B**) available phosphorus (P; mg kg^{-1}), (**C**) available potassium (K; mg kg^{-1}), (**D**) organic carbon (g kg^{-1}), (**E**) pH, (**F**) electrical conductivity (EC; mS m^{-1}), (**G**) cation exchange capacity (cmol(+) kg^{-1}), (**H**) fraction of sand (%), (**I**) fraction of silt (%), (**J**) fraction of clay (%), (**K**) bulk density (Mg m^{-3}), and (**L**) moisture content (g g^{-1}). The boxplot shows the median (horizontal bar), interquartile range (box), 5th and 95th percentiles (whiskers; vertical bars), and outliers (black closed points). Different letters (a, b, c) show the significant differences among the years, as follows: ***: $P < 0.001$, **: $P < 0.01$, *: $P < 0.05$, and n.s.: $P \geq 0.05$.

Fig 4. Relationships of the cation exchange capacity against (A) $\log_{10}$(organic carbon), (B) $\log_{10}$(the fraction of clay), and (C) pH. Open circles represent data in the farmland soils, and closed green circles represent data in the forest soils. ***: $P < 0.001$, *: $P < 0.05$.

Table 1. Soil properties of the farmland and the natural forest. The mean (1 S.D.) values are shown for each property. The significance differences between the sites based on one-way ANOVA are shown as follows; ***: $P < 0.001$, **: $P < 0.01$, *: $P < 0.05$, and n.s.: $P \geq 0.05$.

Soil properties	unit	Farmland soil	Forest soil	df	Sum Squre	Mean Square	*F* value	*P* value	Significance
Kjeldahl nitrogen (N)	g kg^{-1}	1.7 (1.1)	0.9 (0.5)	1	7.02	7.02	7.58	**0.0077**	**
Avalable phosphorus (P)	mg kg^{-1}	6.2 (7.7)	2.0 (1.5)	1	218.69	218.69	4.77	**0.0328**	*
Avalable posassium (K)	mg kg^{-1}	68.8 (76.8)	11 (3.8)	1	40736.56	40736.56	9.11	**0.0037**	**
Organic matter	g kg^{-1}	5.9 (2.7)	14.5 (0.99)	1	896.57	896.57	30.60	**0.0000**	***
pH		5.7 (0.6)	5.9 (0.7)	1	0.31	0.31	0.76	0.3859	n.s.
Electrical conductivity (EC)	mS m^{-1}	5 (6.1)	4 (4.3)	1	3.52	3.52	0.11	0.7443	n.s.
Cation exchange capacity	cmol (+) kg^{-1}	5.7 (2.2)	8.0 (3.4)	1	105.04	105.04	16.11	**0.0002**	*
Sand fraction	%	70.5 (10.4)	69.3 (14.6)	1	18.75	18.75	0.14	0.7085	n.s.
Silt fraction	%	21.5 (8.80)	19.3 (5.7)	1	57.31	57.31	0.86	0.3565	n.s.
Clay fraction	%	8.0 (3.63)	11.4 (8.3)	1	141.63	141.63	4.59	**0.0362**	*
Bulk density	Mg m^{-3}	1.56 (0.141)	1.24 (0.214)	1	1.19	1.19	45.49	**0.0000**	***
Moisture content	g g^{-1}	6.98 (2.909)	5.20 (4.257)	1	37.92	37.92	3.51	0.0657	n.s.

Table 2. The statistical differences among the five years (2016-2020) and among the four locations in each soil property, using repeated one-way ANOVA. Significances are shown as follows, ***: $P < 0.001$, **: $P < 0.01$, and *: $P < 0.05$.

Soil properties	Unit	Expranatory variable	df	Sum Squre	Mean Square	*F* values	*P* values	Significance
Kjeldahl nitrogen (N)	g kg^{-1}	Year	2	19.99	10.00	13.93	**0.000**	***
		Location	3	4.10	1.37	1.90	0.144	n.s
Avalable phosphorus (P)	mg kg^{-1}	Year	2	114.86	57.43	1.00	0.379	n.s
		Location	3	149.88	49.96	0.87	0.482	n.s
Avalable posassium (K)	mg kg^{-1}	Year	2	8894.20	4447.10	1.10	0.347	n.s
		Location	3	8034.00	2678.00	0.66	0.592	n.s
Organic carbon	g kg^{-1}	Year	2	78.71	39.35	24.98	**0.000**	***
		Location	3	26.27	8.76	5.56	**0.013**	*
pH		Year	2	3.15	1.58	11.97	**0.000**	***
		Location	3	0.45	0.15	1.15	0.370	n.s
Electrical conductivity (EC)	mS m^{-1}	Year	2	241.29	120.65	4.04	**0.025**	*
		Location	3	257.73	85.91	2.88	**0.047**	*
Cation exchange capacity	cmol (+) kg^{-1}	Year	2	1.00	0.50	1.28	0.294	n.s
		Location	3	0.51	0.17	0.44	0.730	n.s
Sand fraction	%	Year	2	52.32	26.16	3.96	**0.030**	*
		Location	3	41.69	13.90	2.10	0.153	n.s
Silt fraction	%	Year	2	20.76	10.38	4.98	**0.014**	*
		Location	3	9.42	3.14	1.51	0.263	n.s
Clay fraction	%	Year	2	19.98	9.99	2.82	0.075	n.s
		Location	3	11.57	3.86	1.09	0.391	n.s
Bulk density	Mg m^{-3}	Year	2	0.48	0.24	46.21	**0.000**	***
		Location	3	0.03	0.01	1.90	0.184	n.s
Moisture content	g g^{-1}	Year	2	103.03	51.52	15.93	**0.000**	***
		Location	3	17.92	5.97	1.85	0.192	n.s

Table 3. The results of multiple comparisons (Tukey's honestly significant difference test) among the years and locations. The statistical comparisons were conducted only in soil properties with significant differences ($P < 0.05$) in Table 3. 2016, 2018, and 2020 are the examined years. N: north, E: east, W: west, S: south locations. ***: $P < 0.001$, **: $P < 0.01$, *: $P < 0.05$, and n.s.: $P \geq 0.05$.

Soil properties	Unit	Expranatory variables		Estimated	1 S.D.	*T* values	*P* values	Significance
Kjeldahl N	g kg^{-1}	Year	2018-2016	0.3375	0.299551	1.127	0.835711	n.s.
			2020-2016	1.50625	0.299551	5.028	**0.0001**	***
			2020-2018	1.16875	0.299551	3.902	**0.000822**	***
Organinc carbon	g kg^{-1}	Location	N-E	1.9083	1.1467	1.664	0.4868	n.s.
			S-E	-2.7083	1.1467	-2.362	0.1283	n.s.
			W-E	-0.7833	1.1467	-0.683	0.9781	n.s.
			S-N	-4.6167	1.1467	-4.026	**0.001**	***
			W-N	-2.6917	1.1467	-2.347	0.1328	n.s.
			W-S	1.925	1.1467	1.679	0.4769	n.s.
		Year	2018-2016	-1.4062	0.4437	-3.169	0.0127	*
			2020-2016	-3.1312	0.4437	-7.056	**0.001**	***
			2020-2018	-1.725	0.4437	-3.887	**0.001**	***
pH		Year	2018-2016	-0.1625	0.1283	-1.267	0.75629	n.s.
			2020-2016	-0.6063	0.1283	-4.726	**0.0001**	***
			2020-2018	-0.4438	0.1283	-3.46	**0.00463**	**
EC	mS m^{-1}	Location	N-E	2.9167	2.2298	1.308	0.7305	n.s.
			S-E	-1	2.2298	-0.448	0.9968	n.s.
			W-E	4.8333	2.2298	2.168	0.199	n.s.
			S-N	-3.9167	2.2298	-1.757	0.4247	n.s.
			W-N	1.9167	2.2298	0.86	0.9417	n.s.
			W-S	5.8333	2.2298	2.616	0.0673	n.s.
		Year	2018-2016	-0.6875	1.9311	-0.356	0.999	n.s.
			2020-2016	4.375	1.9311	2.266	0.1604	n.s.
			2020-2018	5.0625	1.9311	2.622	0.0662	n.s.
Sand	%	Year	2018-2016	-2.5563	0.9085	-2.814	**0.0385**	*
			2020-2016	-1.2125	0.9085	-1.335	0.7134	n.s.
			2020-2018	1.3438	0.9085	1.479	0.6156	n.s.
Silt	%	Year	2018-2016	1.2687	0.5104	2.486	0.0946	n.s.
			2020-2016	-0.225	0.5104	-0.441	0.9971	n.s.
			2020-2018	-1.4938	0.5104	-2.927	0.0275	*
Bulk Density	Mg m^{-3}	Year	2018-2016	0.11062	0.02539	4.357	**0.000129**	***
			2020-2016	0.24375	0.02539	9.6	**0.0001**	***
			2020-2018	0.13312	0.02539	5.243	**0.0001**	***
Moisture content	g g^{-1}	Year	2018-2016	-1.9744	0.6358	-3.105	**0.0155**	*
			2020-2016	-3.5825	0.6358	-5.635	**0.001**	***
			2020-2018	-1.6081	0.6358	-2.529	0.0845	n.s.

Table 4. The results of multiple regression analysis for the cation exchange capacity. The cation exchange capacity is the response (dependent) variable, and $\log_{10}$(organic carbon), $\log_{10}$(clay fraction), and pH are explanatory variables. "Estimated" means the slope for each variable. ***: $P < 0.001$, **: $P < 0.01$, *: $P < 0.05$.

Responsible variable	Expranatory variables	Estimated	1 S.D.	T values	P values	Significance
Cation exchange capacity	Intercept	-6.061	2.6758	-2.265	**0.027129**	*
	$\log_{10}$(organic carbon)	3.9304	1.1047	3.558	**0.000738**	***
	$\log_{10}$(clay fraction)	3.1796	1.0334	3.077	**0.00315**	**
	pH	1.0267	0.4426	2.32	**0.023765**	*

Table 5. The concentrations of herbicides (paraquat and glyphosate) in the stream water (upper, middle, and lower streams) and the farmland soil. The herbicide concentrations were examined in the rainy and dry seasons in 2020. The units of the concentrations in stream water and soil are mg l^{-1} (ppm) and mg kg^{-1} (ppm), respectively. “n.d.” means “not detected”.

	Paraquat		Glyphosate	
	Dry season	Rainny season	Dry season	Rainny season
Upper stream	n.d.	n.d.	n.d.	n.d.
Middle stream	n.d.	n.d.	**0.5**	n.d.
Lower stream	n.d.	n.d.	n.d.	n.d.
North_Subplot 1	n.d.	n.d.	n.d.	**1.5**
North_Subplot 2	n.d.	n.d.	**1.3**	n.d.
North_Subplot 3	n.d.	n.d.	n.d.	**5.0**
North_Subplot 4	n.d.	n.d.	n.d.	n.d.
East_Subplot 5	n.d.	n.d.	n.d.	**1.5**
East_Subplot 6	n.d.	n.d.	n.d.	n.d.
East_Subplot 7	n.d.	n.d.	n.d.	n.d.
East_Subplot 8	n.d.	n.d.	n.d.	n.d.
West_Subplot 9	n.d.	n.d.	**1.3**	n.d.
West_Subplot 10	n.d.	n.d.	n.d.	**1.5**
West_Subplot 11	n.d.	n.d.	n.d.	n.d.
West_Subplot 12	n.d.	n.d.	n.d.	n.d.
South_Subplot 13	n.d.	n.d.	n.d.	n.d.
South_Subplot 14	n.d.	n.d.	n.d.	**2.5**
South_Subplot 15	n.d.	n.d.	**2.5**	n.d.
South_Subplot 16	n.d.	n.d.	n.d.	n.d.

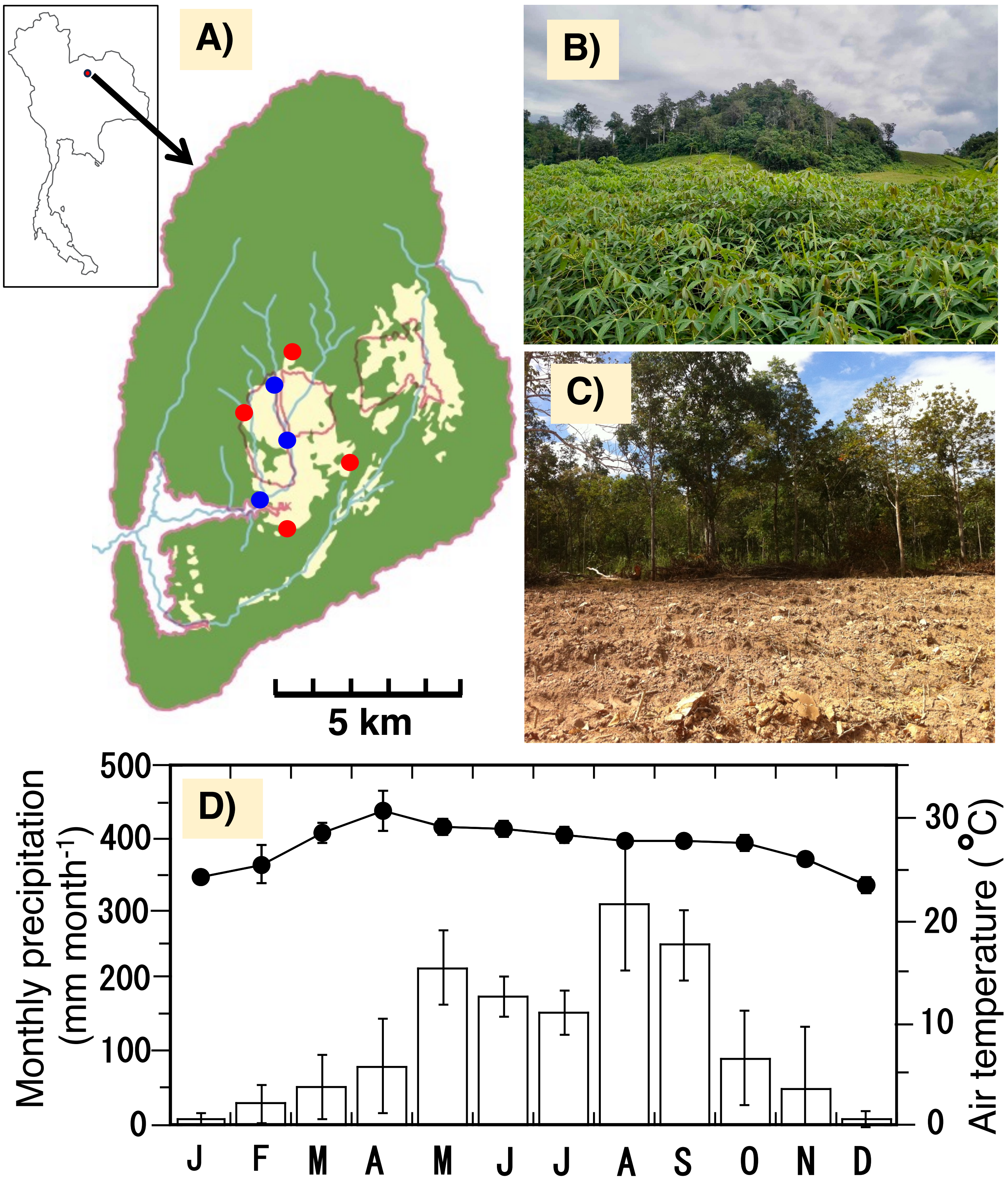
A)
B)
C)
5 km
D)
Monthly precipitation (mm month-1)
Air temperature (°C)
0
100
200
300
400
500
0
10
20
30
J
F
M
A
M
J
J
A
S
O
N
D

Figure 2

Click here to access/download;Figure;Fig2_Ishida.pdf

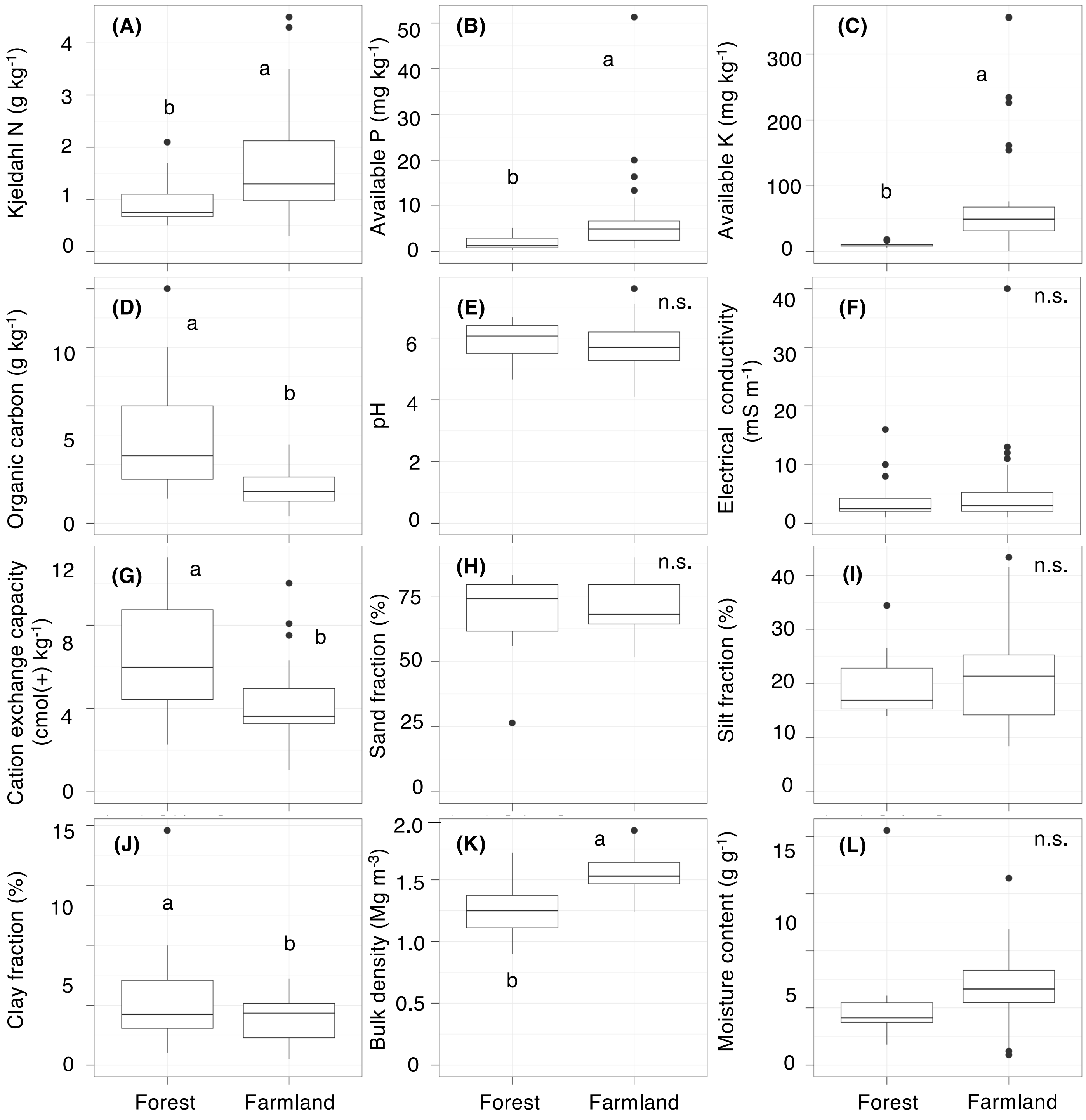

Figure 3

Click here to access/download;Figure;Fig3_Ishida.pdf

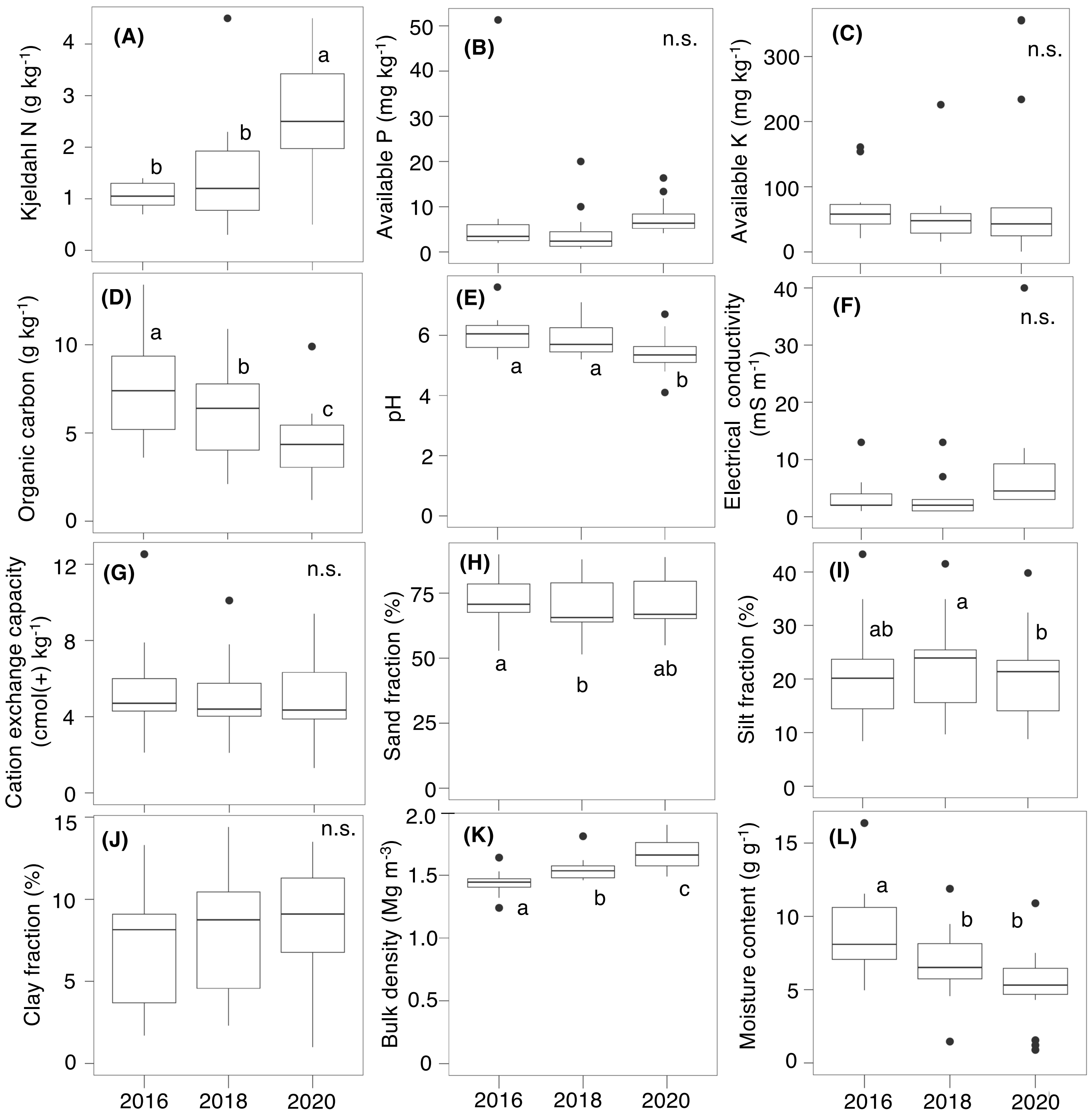

Click here to access/download;Figure;Fig4_Ishida.pdf

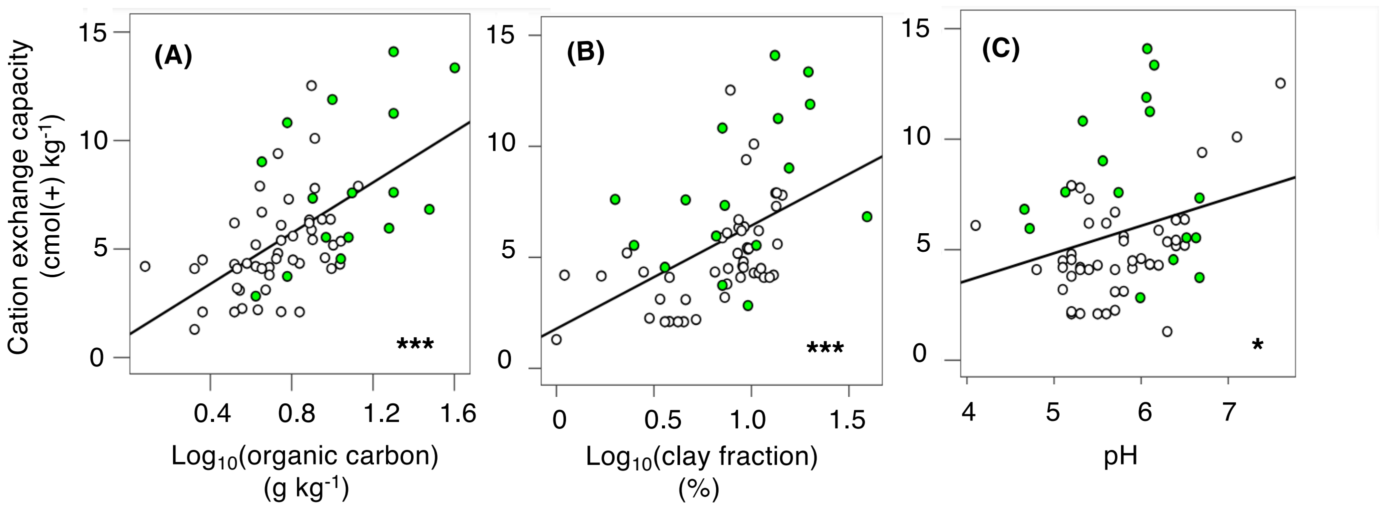

Click here to access/download
Supporting Information
Farmland_S1.xlsx

Click here to access/download
Supporting Information
ForestSoil_S2.xlsx

Click here to access/download
Supporting Information
S1 Fig.pdf

Title: Soil degradation and herbicide pollution by repeated cassava monoculture within Thailand's conservation region

Short Title: Soil degradation and herbicide pollution in cassava monoculture

Authors: Ananya Popradit (ORCID 0000-0002-1546-7813) [1*], Yutthana Nakhokwik[1], Marcel Robischon[2], Shin-Taro Saiki (ORCID 0000-0002-3685-2665) [3], Jin Yoshimura (ORCID 0000-0003-1610-1386) [4,5,6], Arichai Wanasiri[7] and Atsushi Ishida (ORCID 0000-0003-0620-5819) [8*]

Affiliations:

1 Doctor of Philosophy Program in Environmental Studies, Valaya Alongkorn Rajabhat University under Royal patronage, Pathum Thani 13180, Thailand.

2 Faculty of Life Sciences, Albrecht Daniel Thaer Institute for Agricultural and Horticultural Sciences, Department of Agroecology, Humboldt University of Berlin, Berlin D-10099, Germany.

3 Forestry and Forest Products Research Institute, Matsuno-sato, Tsukuba, Ibaraki 305-8687, Japan.

4 Institute of Tropical Medicine, Nagasaki University, Sakamoto, Nagasaki, Nagasaki 852-8523, Japan.

5 Faculty of Science, Tokyo Metropolitan University, Minami-Osawa, Hachioji, Tokyo 192-0397, Japan.

6 The University Museum, The University of Tokyo, Hongo, Bunkyo, Tokyo 113-0033, Japan.

7 Department of National Parks, Wildlife and Plant Conservation. Ban Kho Subdistrict, Non Sang District, Nong Bua Lamphu 39140, Thailand.

8 Center for Ecological Research, Kyoto University, Otsu, Shiga 520-2113, Japan.

Correspondence Authors:

Ananya Popradit: ananya-toon@hotmail.co.th

Atsushi Ishida: atto@ecology.kyoto-u.ac.jp

Data Availability Statement: All raw data are within the paper and its Supporting Information files.

Funding: This study was supported by funding provided from the Japan Society for the Promotion of Science (No. 16H02708, 23KK0119, 24K03129). The funders had no role in study design, data collection and analysis, decision to publish, or preparation of the manuscript.

Competing interests: The authors declare no competing interests.

Abstract

In a national park in Northeast Thailand, agricultural land has been converted from natural forest by small-scale farmers for cassava agriculture. We hypothesise that long-termed cassava monoculture leads to soil degradation. To test the hypothesis, we conducted a five-year (2016-2020) study on the physical and chemical properties of soil in cassava farmland, and also examined the soil properties of its adjacent natural forests, as a control. Soil nitrogen, available phosphate and potassium, and bulk density were significantly higher in the farmland than in the forest, whereas cation exchange capacity, organic carbon, and clay fraction showed the opposite trend. The cation exchange capacity was positively correlated to the fraction of clay and the organic carbon within soil. These data indicate that the farmland soil was irreversibly degraded by the conversion from forest to cassava fields, through rapid decreases in cation exchange capacity, organic carbon, and the fraction of clay. For the farmland, soil nitrogen and bulk density increased significantly, whereas pH and organic carbon decreased significantly over the five years, indicating that these soil properties were gradually altered by repeated agricultural activities, such as fertilizer application and trampling. The use of glyphosate and paraquat herbicides is prohibited within national parks in Thailand. However, in 2020, glyphosate was detected in farmland soil (up to 5.0 mg kg^{-1}) during both the rainy and dry seasons, and glyphosate (2.5 mg l^{-1}) was detected in stream water from the farmland during the dry season. The use of glyphosate in the farmlands was thus suspected at least in 2020. Soil degradation and herbicide pollution may carry a high risk of causing irreversible changes in terrestrial ecosystems. We discuss the root causes of this issue from perspectives of agricultural production, economy, and the environmental impact, and propose effective policy measures.

Introduction

The global agricultural land area increased by 102 million hectare during the first two decades of the 21st century, corresponding to 9% of the agricultural land area in 2003[1]. During the 16 years from 2003 to 2019, the per capita agricultural land area decreased by 10% owing to population growth, while the per capita net primary production of agricultural land area increased by 3.5% owing to the enhancement and intensification of agriculture [1]. The increase in crop yields per capita and land area has resulted from the modernization of agriculture. Modernization relies on the development of agricultural facilities and cultivation techniques, development and utilization of agricultural machinery, introduction of improved crop varieties with higher yields, and long-term input of chemical fertilizers and pesticides in farmland. In particular, agricultural modernization is based on large-scale cultivation using a single-crop variety with a high yield (i.e., monoculture).

However, in homogeneous agriculture, diseases and pests are more likely to occur than in traditional agriculture [2]. In such monocultural farmlands, the heavy use of chemically synthesized pesticides and fertilizers is usually required for effective pest control and enhancement of crop yields [3]. In Thailand, the importation of herbicides increased by 10% over a four-year period, rising from 53615 tons in 2009 to 60232 tons in 2012, with glyphosate being the most imported herbicide, both in terms of cost and quantity [4]. Nevertheless, herbicides and pesticides have the potential to cause significant negative impacts on humans (including children), livestock, and the environment if mishandled [5-8]. Continuous attention is thus needed to evaluate and improve regulatory systems and their social implementation. However, in many developing countries, the effectiveness of regulatory systems is often low [6, 9]. Because of its broad effectiveness on both annual and perennial plants (including

various crops), glyphosate is popular in Thailand, particularly for effective weed control before planting, even in farmlands [4].

Cassava (*Manihot esculenta* Crantz) is well-suited to tropical and subtropical regions with warm temperatures [10]. This crop plant can grow even in nutrient-poor soil, drought, and heat environments [11] and has significant economic importance for local farmers in tropics [12-15]. Monocultural farming is a common practice worldwide for cassava cultivation. Such monocultural agroecosystems have various risks of soil degradation, including erosion on steep-slope farms, soil compaction and crusting, structural deterioration, flooding, organic carbon loss, microbial decline, salinization, and on-site and off-site damage [16]. Cassava roots penetrate only up to 0.5 m deep into the soil, but can extend 1-2 m horizontally depending on the planting density, contributing to the suppression of soil erosion [17]. However, some reports have showed that the loss of farmland soil is significant especially when cassava roots are harvesting [18-20]. On the effects of soil nutrients, cassava extracts significantly less nitrogen and phosphorus from the soil than most other crops while extracting similar amounts of potassium (K) [12]. Consequently, additional fertilization is needed to maintain soil fertility when cassava is continuously cultivated on the same land, especially K [13, 18, 21-23], phosphorus [18, 24, 25], and nitrogen [24], possibly leading to the overuse of fertilizers and an imbalance of nutrient levels within the farmland soil [26]. While once thought to be environmentally friendly, even low-input crops like cassava are now being recognized as contributors to cycles of environmental degradation that endanger future food production [15].

In terms of above-ground environmental factors, including air temperature, solar radiation, and precipitation in Northeast Thailand, the potential production of cassava can be further increased by adopting adequate water management, especially by

managing and expanding agricultural land in the mid-highlands [27]. However, we do not have sufficient information regarding soil factors or adequate management based on environmental conservation. In particular, long-term monitoring of soil quality in cassava farmlands is still rare. Here, we hypothesise that long-termed cassava cultivation leads to soil degradation. Additionally, we examined whether herbicides prohibited within Thailand's national parks were used. To sustainably obtain sufficient cassava yields, it is necessary to understand the extent of soil degradation caused by repeated cassava monocultures, and to know the actual impact of the long-term use of chemical fertilizers and pesticides, including herbicides, on soil and water. In the present study, we identify which soil properties incur irreversible damage due to agricultural activities, and furthermore, provide evidence to raise suspicions about the agricultural use of banned glyphosate.

Materials and Methods

Study site and method overview

This research was conducted in a cooperative research project of the Department of National Parks Wildlife and Plant Conservation, Thailand (Permission No. 5810701, 5810107, 5921601). All procedures in this study were carried out in accordance with the relevant guidelines of Valaya Alongkorn Rajabhat University. Phu Kao-Phu Phan Kham National Park (PKNP) is a national park located on the Khorat Plateau with sandy soil in Northeast Thailand. The study site (16°55'48.76"N, 102°27'40.12"E) is located in Phu Kao within this national park (Fig 1). Since 1998, the natural forests within this national park have been designated as protected areas for natural conservation. The Khorat Plateau is primarily composed of nutrient-poor sandy soil. On

this plateau, dry deciduous forests develop naturally [28, 29] and are composed of canopy trees that have adapted well to the oligotrophic sandy soils [30]. During the last nine years from 1991 to 2020, the yearly precipitation was 900-1300 mm (1252 mm on average), and the yearly mean air temperature was 21.1-32.5°C (26.0°C on average) at approximately 22.7 km southeast of the study site (based on the Thai Meteorological Department). There is a distinct dry season from November to January (Fig 1D).

In Phu Kao, there are three villages (Dong Bak, Wang Mon and Chai Mongkala) surrounded by agricultural lands, which are further enclosed by natural forests. Cassava monoculture has been predominantly carried out on agricultural land, which has been converted from natural forests since the 2000s. Although the boundary line between farmland has and protected natural forests was established in 2010 by the Thai government, agricultural land had expanded into protected forest areas beyond the boundary line during the successive five years (2011 to 2015) (Fig 1A). During the five years from 2016 to 2020, soil properties were examined at four locations (north, east, south, and west of the main village) in the cassava farmland. To further clarify the agricultural impact on soil, we examined the soil properties of the nearest forests at each of those four locations. The combination of comparisons between farmland and forest soils and long-term monitoring of farmland soil will clarify which soil properties are largely affected by agriculture.

Soil and water sampling

Soil samples were collected from at a depth of 15 cm from the soil surface, using a drop-hammer sampler equipped with double cylinders (ANS Inc., American Falls, ID, US). The size of inner cylinder was 4.5 cm in diameter and 15.2 cm in length (approximately 242 cm^3 in volume).

The farmland soil was collected after crop harvesting in the dry season, when the soil nutrient levels would be lowest in each growing season. Soil sampling was conducted at four locations (the north, east, south and west of the main village) (Fig 1A), and four subplots were subsequently set in each location (16 subplots in total). The positions of the 16 subplots were determined using a Global Positioning System (GPS; Garmin 60Csx, Garmin, Olathe, KS, USA), and the soil (0-15 cm thickness below the ground surface) was collected at the same positions three times (2016, 2018, and 2020). Furthermore, to compare the soil properties between the farmland and forest soils, we investigated the soil properties using soil samples collected from the nearest natural forests at the four locations during the dry season on November 15, 2020.

For nature conservation, the use of glyphosate and paraquat within national parks has been prohibited by the Thai government. During the dry season, stream water was blocked in the lower stream to obtain irrigation water. Because of the halted waterflow, pollutant accumulation was predicted during this season. To examine the possibility of herbicide pollution within farmland ecosystems, paraquat and glyphosate were tested in soil and stream water during the rainy (June 6) and dry seasons (November 18) in 2020. Farmland soils were collected from the 16 subplots. The water of the main stream flowing through the agricultural area was collected at three locations (upper, middle, and lower streams) with one sample taken at each location (Fig 1A).

Soil analysis

To examine the impact of long-term agricultural land use on farmland soil in Phu Koa, we examined 10 soil properties, soil texture (the fractions of sand, silt, and clay), cation exchange capacity, bulk density, pH, Kjeldahl nitrogen (N), available/exchangeable phosphorus (P), available/exchangeable potassium (K), organic carbon, electrical

conductivity (EC), and soil moisture contents, in monocultural cassava farmland over a five-year period (2016-2020). For soil physical properties, soil texture (dry mass %), bulk density (Mg m^{-3}), and soil moisture content (g g^{-3}) were examined. All raw data for the farmland and forest soils (0-15 cm depth) are represented in Supplementary Information, S1 and S2 Tables, respectively.

To examine the soil texture, the collected soil was sieved with a 2-mm grid mesh, and subsequently air-dried. The volumetric composition of sand (> 20 μm in diameter), silt (2-20 μm) and clay (< 2 μm) in the soil samples were determined by the hydrometer method [31, 32] using an ASTM specific gravity hydrometer (Type 152H). The moisture contents were determined from air-dried soil samples as follows:

Soil moisture content = (soil wet mass – soil dry mass)/soil dry mass (1).

To examine the soil bulk density, the soil contained within the inner cylinder (242 cm^3) of the drop-hammer sampler was subsequently dried in an oven at 105°C for approximately 18-24 hours. The values of bulk density (Mg m^{-3}) were determined from the ratio of soil dry mass to the inner cylinder volume.

For chemical properties, the collected soils were air-dried, ground, and sifted through a 2-mm grid mesh before chemical analysis. The pH values in the soil solution extracted with distilled water were measured using a pH meter [33]. Soil N concentration (g kg^{-1}) was determined using the Kjeldahl method based on a wet combustion [34]. The available P concentration (mg kg^{-1}) was extracted with Bray II solution (0.03 N NH_4F/0.1 N HCl), and the concentration was colorimetrically examined with a spectrophotometer (U-2000, Hitachi Ltd., Tokyo, Japan) based on the reaction with ammonium molybdate and the development of molybdenum blue colour [35]. The available K concentration (mg kg^{-1}) was extracted using 1 molar NH_4OAc solution (pH 7.0), and the extracted K content was colorimetrically examined with a spectrophotometer (XP Flame Photometer,

BWB Technologies Ltd, Newbury, UK) [36]. The soil organic carbon content (g kg^{-1}) was examined using the Walkley-Black chromic acid wet oxidation method, and the oxidizable matter in the soil was oxidized using a 1 N potassium dichromate ($K_2Cr_2O_7$) solution [37]. Electrical conductivity (EC; mS m^{-1}) was measured with an EC meter (Soils Thermal Conductivity Meter, Model MK-TDS210-B, Hangzhou Asmik Sensors Technology Co., Ltd., Zhejiang, China) using a solution extracted with distilled water (1:5). The cation exchange capacity (cmol(+) kg^{-1}) was examined using the ammonium saturation method by saturating the soil with a solution of ammonium acetate (NH_4OAc, 1 M, pH 7) [38]. NH_4^+ replaces the cations that are absorbed by all of the clay micelles in the soil. The amount of NH_4^+ was determined by distillation after it was replaced by Na^+ from NaCl, which is a direct replacement method.

Assessing herbicide pollution

Water was collected from the main stream of the farmland. We added 0.5 ml of formic acid to 50 ml of the water samples and purified through the Osais HLB solid-phase extraction cartridge (Waters Corporation, MA, USA). The herbicide concentration in the water was examined as follows. We added 20 ml of 1% formic acid water/methanol to 20 g of soil sample (1:1 volume), and the mixture was shaken with a vortex mixer for 5 min. The samples were left to settle for 30 minutes and then centrifuged at 5,000 rpm for 5 min. A 5 ml sample of the supernatant was purified through the Osais HLB solid-phase extraction cartridge. To examine the paraquat and glyphosate concentrations, acetonitrile was used for one-step extraction and dewatering of the samples with $MgSO_4$ and purification with dispersive-solid-phase extraction (dispersive-SPE). Organic acids, other constituents, and excess water were removed using PSA (primary and secondary amine), C18-EC (end-capped) and $MgSO_4$, respectively [39]. Chemical

analyses were conducted at 4°C for 24 h. The paraquat and glyphosate concentrations in the soil and water were examined using high-strength liquid chromatography (UltiMate 3000 HPLC, Thermo Fisher Scientific, MA, US).

Statistics

For each soil property, significant differences between the agricultural and forest soils were examined using the pooled data from each site with one-way analysis of variance (ANOVA) (Table 1). The statistical variations among the five years (2016-2020) and among the four locations in each soil property were examined using repeated one-way ANOVA (Table 2). For soil properties with significant differences ($P < 0.05$) in Table 2, multiple comparisons using Tukey's honestly significant difference (HSD) test were conducted between the years and the locations (Table 3). To evaluate the contribution of the organic carbon, the fraction of clay, and pH to the cation exchange capacity, multiple correlation analysis was conducted, as follows: the cation exchange capacity was the dependent variable, and $\log_{10}$(organic carbon), $\log_{10}$(clay fraction), and pH were explanatory variables, employing a GLM assuming a Gaussian error distribution with an identity link function (Table 4).

All statistical analyses were conducted using R software [40] and RStudio (ver. 2023.06.1; RStudio, Boston, MA, USA). Statistical significance was recognized by $P < 0.05$ (***: $P < 0.001$, **: $P < 0.01$, *: $P < 0.05$, n.s.: $P \geq 0.05$).

Results

Soil properties

All raw data for the farmland and forest soils (0-15 cm depth) are represented in Supplementary Information, S1 and S2 Tables, respectively. The soil properties of

farmland showed significant differences with those of adjacent forest soils in some parameters (Fig 2, Table 1). For the soil texture, no significant differences were found in the fractions of sand and silt, whereas the fraction of clay was significantly lower in the farmland soil than in the forest soil. The sand fractions in the farmland and forest soils were 70.5% and 69.3%, respectively, indicating that the soil within this region was sandy because of the sandstone bedrock. Regarding soil chemical properties, Kjeldahl nitrogen (N; sum of organic N and ammonia N), available phosphorus (P), and available potassium (K) were significantly higher in the farmland soil than in the forest soil, whereas cation exchange capacity and organic carbon were significantly lower in the farmland soil. The average concentrations of N, P, and K in the farmland soil were 1.9 times, 3.1 times, and 6.3 times higher than those in the forest soil, respectively, and the cation exchange capacity of the farmland soil decreased by 71% compared with that of the forest soil. No significant differences were found in pH and EC between farmland and forest soils.

The annual variations in soil properties in the farmland over the five years (2016-2020) are shown in Fig 3 (see Tables 2, 3 for statistics). No significant yearly variations were found in available P, available K, cation exchange capacity, and the clay fraction. Soil Kjeldahl N and bulk density in 2018 and/or and 2020 increased significantly from those in 2016, whereas soil organic carbon, pH, and moisture content decreased significantly. Among the locations, no significant variations were not found for all soil properties, except for the organic contents between the south (S) and north (N) sites (Table 3, S1 Fig).

Both cation exchange capacity and the fraction of clay were significantly lower in the farmland than in the forest (Fig 2), and no yearly variations were found in these soil properties (Table 2). Assuming that the soil before deforestation was similar to the

examined forest soil, the current data showed that the conversion to cassava fields resulted in a rapid decrease in cation exchange capacity and clay fraction within several years before starting this observation (at least the period from 2011 to 2016), followed by a delayed decrease in organic carbon (Fig 3D). The significant decrease in pH was found in only 2020 (last year) (Fig 3E). Over all, the cation exchange capacity was significantly and positively correlated to $\log_{10}$(organic carbon), $\log_{10}$(clay fraction), and pH (Fig 4). The results of the multiple correlation analysis indicated that the contribution to cation exchange capacity increased in the following order: pH, the fraction of clay, and organic carbon (Table 4).

Paraquat and glyphosate pollution

In 2020, we assessed glyphosate and paraquat in farmland soil and the water of the main stream in farmland (Table 5). Paraquat was undetectable in both the soil and stream water. Unfortunately, glyphosate in the farmland soil was detected at levels of up to 5.0 mg kg^{-1} in both the rainy and dry seasons throughout at all locations. In the main stream, glyphosate (2.5 mg l^{-1}) was observed in the water of the middle stream in the dry season, when waterflow had stopped. In the rainy season, glyphosate was not detected in the stream water, probably because of continuous waterflow. It was confirmed that glyphosate was widely used within farmlands, at least in 2020.

Discussion

Soil degradation

Significant differences in soil health were found between the forest and the cassava farmland soils. The cation exchange capacity is a measure of the ability to adsorb and retain cations in soil, showing that as the values increase, the soil can retain more

nutrient ions. Both the cation exchange capacity and the clay fraction of forest soil was 1.4 times higher than that of farmland soil (Fig 2), and no significant variations were found across years for the farmland soil (Table 2). Soil organic of forest soil was 2.5 times higher than that of farmland soil, and the soil organic contents of farmland decreased yearly (Fig 2). The conversion from forest to cassava fields resulted in rapid decreases in cation exchange capacity and clay fraction, followed by delayed decreases in organic carbon and pH. Usually, the cation exchange capacity is affected by clay fraction [41 Ohta 2000] and organic contents and pH [42 Haynes 1998, 43 Jarecki 2005]. The cation exchange capacity significantly decreased with decreasing the fraction of clay, the organic carbon, and pH (Fig 4). These facts indicate that the conversion from natural forests to farmland results in rapid and irreversible soil degradation through a decline in cation exchange capacity, associated with decreases in clay fraction, organic carbon and pH.

Soil nutrients

Cassava can grow even in nutrient-poor soil, but to obtain sufficient yields, it is necessary to apply fertilizers containing many elements including K, N, and P [13, 18, 21-25]. In Thailand, non-fertilizer treatment over a period of 15-30 years induces a decrease of 30-40% from the initial cassava yield per unit area [12]. For the yearly change of farmland soil properties, Kjeldal N and bulk density increased significantly, whereas pH, organic carbon, and moisture content decreased significantly (Fig 3). These yearly changes would be due to repeated agricultural activities, and farmers have been continuously applying excess N fertilizer to maintain sufficient cassava yields. The increase in bulk density indicates soil compaction caused by repeated agricultural trampling, possibly reducing cassava growth through inhibition of root penetration and

nutrient uptake [44, 45]. The average concentrations of Kjeldal N, available P, and available K in the farmland soil were 1.9 times, 3.1 times and 6.3 times higher than those in the forest soil, respectively (Fig 2). The increase in soil N and acidification could be due to the overuse of chemical fertilizers, compensating for nutrient loss throughout the repeated crop harvesting with artificial fertilization. It has known that additional K fertilization leads the increases in the number and diameter of cassava roots [23], and soil P and N are the major limiting factors of plant growth, particularly in sandy oligotrophic soil of Thailand [30].

Excess chemical N fertilizer leads to soil acidification, which is a major global issue of sustainable development for agricultural ecosystems [46, 47]. Although no significant variations were not found in pH values between the farmland and forest soils (Table 1), the pH values of 2020 (5.4 in average) were significantly lower than those of 2016 (6.0 in average) and 2018 (5.8 in average) for the farmland soil (Fig 3). Aluminum (Al) is the third most abundant element in the Earth's crust, following oxygen and silicon, and it is universally present in soils [48]. Al in soil begins to leach out at pH below 5.5 and then Al^{3+} ions become the predominant form at pH below 4.5, inducing the inhibition of root elongation in plants [49]. Cassava is less sensitive to acid soils than grain crops, such as maize (*Zea mays* L.), on Oxisoles, Ultisols, and Alfisols of tropics [50]. However, the prolonged exposure of cassava roots to high Al^{3+} levels gradually and continuously promotes proton release from their roots and leads to the dissolution of Al^{3+} ions and soil acidification, resulting in high metal toxicity and low P availability [47]. Therefore, it is recommended to keep the soil pH below 5.5 even in cassava, to mitigate aluminum dissolution and the toxicity caused by high Al^{3+} ions [47].

In addition, the overuse of chemical fertilizers rapidly reduces soil organic carbon due to soil acidification and microbial decline [51]. At the present study site, the organic carbon of forest soil was 2.5 times higher than that of farmland soil on average (Fig 2). Soil organic carbon plays a crucial role in governing not only cation exchange capacity [42, 43] but also the physicochemical reactions that regulate the availability of micronutrients (such as Zn, Cu, Fe, Mn, B, and Mo) [52]. The cation exchange capacity is usually defined as the amount of positive charge that can be exchanged per soil mass. The values (mean ± 1 S.D.) of cation exchange capacity in the forest and farmland soils were 8.0 ± 3.4 and 5.0 ± 2.2 cmol (+) kg^{-1}, respectively (Fig 1). Not only these values of cation exchange capacity but also those of N and P concentrations at this study site are extremely low and comparable to the values of degraded or sandy soil in East Kalimantan, Indonesia [41], indicating that the Khorat Plateau (the present study site) originally had oligotrophic soil because of the sandy soil [30].

Such oligotrophic soil and soil degradation advanced by the cassava monoculture resulted in low crop yields, consequently increasing the need for chemical fertilization. Tropical soils generally lack resilience once degraded [53]. Soil degradation and chemical pollution thus pose a high risk of making ecosystems irreversibly unable to return to their original state. The nitrogen remaining in the soil undergoes transformation into nitrate ions (NO_3^-) within the soil, and NO_3^- readily dissolves in water. Nitrogen loading in the nutrient-poor sandy soil of natural forests may have unexpected effects on canopy trees that have adapted to oligotrophic soil for a long time [30]. The soil degradation caused by repeated cassava monocultures can thus be a major driver of negative feedback in local-scale environmental conservation. Furthermore, dissolved NO_3^- facilitates leaching from the soil into groundwater and river water.

Eutrophication can occur when a significant influx of nutrients occurs in rivers, lakes, and seas.

Herbicide use

The use of pesticides has been increased worldwide, thereby increasing the risk of environmental exposure. Although the direct toxicity of glyphosate to humans is low, the risk of inducing cancer remains controversial and several glyphosate hotspots in the soil have been detected across the European Union [54]. In Thailand, almost all insecticides and herbicides are imported [6]. Since June 1, 2020, the use of chlorpyrifos and paraquat was prohibited, whereas the application of glyphosate was permitted under specific restrictions governed by the requirement of the maximum residue level (MRL). Since June 30, 1998, the use of all herbicides has been prohibited in all forest reserves and their watershed areas in Thailand, to prevent hazards and contamination in natural water sources due to spread by wind and water erosion [55-57]. Several recent experiments exposing small mammals (rats) to glyphosate have shown that even low concentrations of glyphosate can cause significant damage to offspring in successive generations (F2 and F3) rather than the parental generation (F1) because of abnormal DNA methylation of the germline (e.g., sperm) [58-60].

Although the half-life of glyphosate in soil ranges between 2 and 197 days, the illegal use of glyphosate in this cassava farmland was recognized even within the national park at least in 2020 (Table 5). To sustain the increase in family members, the use of banned herbicides would provide an easy and cost-effective solution for local farmers to increase cassava yields. Nevertheless, investment in chemical fertilizers accounts for 53% of their production costs [28]. Herbicides, such as glyphosate, are used worldwide to control weeds. Although Thailand has not yet approved genetically

modified crops (GOC) that can tolerate glyphosate [4], glyphosate-resistant weeds have been rapidly expanding worldwide owing to the excessive use of GOC and glyphosate [61, 62]. The development of alternative herbicides has also been attempted [63, 64], and low-cost generic pesticides and herbicides are readily available worldwide. However, a decrease in pesticide costs may result in an increase in the frequency of its application. Furthermore, because of the quality variations among generic pesticides depending on the manufacturing process and raw materials, it is crucial to ensure their effectiveness and safety through regulation and management on a global scale [9].

Socioecological perspectives

This case study from Northeast Thailand presented here is a common issue faced by developing countries worldwide generally face. To address this problem and achieve sustainable socioecosystems, it is necessary to comprehensively consider the root causes of this issue, including population growth in tropical regions, agricultural production, and the economy, and their impacts on the environment.

Over the last decade, the growth of the human population has resulted in the remarkable expansion of cassava farmland by local-scale farmers, beyond the boundary line between farmland and protected natural forests that set by the Thai government in 2010 (see Fig 1) [37]. Significant tree species loss was also found within the protected natural forest beside the farmland [28]. The processes of urbanization and agricultural development have had a significant impact on not only the natural environment but also on local socioeconomic factors, including the labour force, education expenses, employment opportunities, household commodities, and vehicles [65-67]. In Thailand, the economic growth rate reached approximately 7.8% per year during the period of the Seventh National Economic and Social Development Plan (1992-1996), whereas the

annual growth rate in the country's agricultural sector during the same period was only 2.5% [67]. Certainly, the rate of poverty reduction is closely related to the economic growth rate while facing a consistent increase in income inequality [68]. The proportions of farmland area and forests are 40.7% and 25.3%, respectively, of the country's area (based on the Ministry of Agriculture and Cooperation), and 44.6% of the workforce is involved in the agricultural sector, but contributes only 7.2% of the country's GDP (National Economic and Social Development Board data). Although approximately 67% of the human population resides in rural areas in Thailand (1995-2000), many agricultural lands have been converted into industrial zones, luxury resorts, and towns, resulting in an overall shortage of agricultural land [67]. Nevertheless, a large proportion of the population in Thailand is still engaged in agriculture, which serves as a significant source of income.

To ensure food security and production, the development of new agroecosystems based on environmental conservation is required in the tropics overall. A transition from monocultural cassava cultivation to new farmland use, including the idea of traditional agricultural systems, is necessary. Although traditional slash-and-burn farming has only low productivity, additional chemical fertilizers and herbicides are not needed, having the advantages of low cost and low soil degradation risks [53, 69]. A recent meta-analysis showed that crop diseases decrease and crop yields increase with increasing diversity in intraspecific cultivar mixtures [2]. Another meta-analysis of cassava-based agroecosystems indicated that interspecies cropping had provides positive effects on several key ecosystem services, including pest suppression, disease control, and soil- and water-related services [16]. Experimentally, mixed cropping systems, including intercropping and crop rotation systems, have been shown to play a significant role in increasing cassava yields [17, 70-72]. Thus, diversified agriculture

can thus play a significant role in ensuring a balance between productivity, crop resilience, and environmental health [73, 74]. Furthermore, the use of organic mulch as an alternative to plastic mulch and chemical herbicides is effective for maintaining crop yields through adequate or moderate weed control [75] and soil improvement effects [42], potentially reducing the use of chemical fertilizers and herbicides [76, 77].

Nevertheless, in social implementation, political systems that support income and promote diverse crop cultivation are important, particularly for small-scale farmers. Enhancing ecological agricultural systems, using multiple crops with various adaptive characteristics will be effective not only for maintaining human health and well-being but also for strengthening resilience to climate change, thereby improving food security in developing countries [15, 73]. Adopting agricultural systems that promote ecosystem services enhances resistance not only to pest and disease control but also to climate change, reducing the risk of decreased crop production due to climate change and contributing to the development of a resilient society.

Conclusion

The comparison of farmland and forest soils, coupled with long-term monitoring of farmland soil, effectively described the characteristics of soil degradation resulting from repeated cassava monoculture. The significant decreases in clay fraction, organic carbon, and cation exchange capacity in the farmland soil indicate that the conversion from forest to farmland leads to rapid and irreversible soil degradation. For the yearly change in the farmland, soil N and bulk density were increasing, whereas organic carbon and pH were decreasing significantly. Although these soil properties may be relatively quickly recovered by cessation of soil fertilization and repeated agricultural trampling, these agricultural activities will be required to maintain sufficient cassava

yields. Because the farmland is located within a national park, the use of herbicides is prohibited. However, we provide evidence of the illicit use of herbicides and the unauthorized expansion of agricultural activities into protected natural forests. For nature conservation, it is necessary to establish political systems that support local farmers financially, promote the transition to diverse crop cultivation, and encourage the use of organic mulch.

Supporting information

S1 Table. Raw data for the farmland soil properties. (XLSX)

S2 Table. Raw data for the forest soil properties. (XLSX)

S1 Fig. Variations in soil properties among the locations for the farmland. (PDF)

Acknowledgements

We wish to thank Mr. Y. Kanzaki for drawing Figure 1. This research was a cooperative research project of the Department of National Parks Wildlife and Plant Conservation, Thailand.

Author Contributions

Conceptualization: Ananya Popradit, Marcel Robischon, Arichai Wanasiri

Formal analysis: Shin-Taro Saiki, Ananya Popradit

Project administration: Ananya Popradit, Arichai Wanasiri

Investigation: Ananya Popradit, Yutthana Nakhokwik

Writing – original draft: Atsushi Ishida, Ananya Popradit, Jin Yoshimura

References

1. Potapov P, Turubanova S, Hansen MC, Tyukavina A, Zalles V, Khan A, et al. Global maps of cropland extent and change show accelerated cropland expansion in the twenty-first century. Nature Food. 2022; 3: 19-28. doi.org/10.1038/s43016-021-00429-z

2. Reiss ER, Drinkwater LE. Cultivar mixtures: a meta-analysis of the effects of intraspecific diversity on crop yield. Ecol Appl. 2018; 28: 62-77. doi.org/10.1002/eap.16292018

3. Lithourgidis AS, Dordas CA, Damalas CA, Vlachostergios DN. Annual intercrops: an alternative pathway for sustainable agriculture. Aust J Crop Sci. 2011; 5: 396-410.

4. Suvittawat A. Gyphosate market in Thailand: opportunities and challenges. Inf Manag Bus Rev. 2014; 4: 207-211. doi.org/10.22610/imbr.v6i4.1116

5. Bai SH, Ogbourne SM. Gyphosate: environmental contamination, toxicity and potential risks to human health via food contamination. Environ Sci Pollut Res. 2016; 3: 18988-19001. doi.org/10.1007/s11356-016-7425-3

6. Laohaudomchok W, Nankongnab N, Siriruttanapruk S, Klaimala P, Lianchamroon W, Ousap P, et al. Pesticide use in Thailand: Current situation, health risks, and gaps in research and policy. Hum Ecol Risk Asses. 2020; 27: 1147-1169. doi.org/10.1080/10807039.2020.1808777

7. Berkal MA, Nardinm C. Pesticide biosensors: trends and progresses. Anal Bioanal Chem. 2023; 415: 5899-5924. doi.org/10.1007/s00216-023-04911-4

8. Grammenou A, Petropoulos SA, Thalassinos G, Rinklebe J, Shaheen SM, Antoniadis V. Biostimulants in the soil-plant intertace: Agro-environmental implications - A

review. Earth System Environ. 2023; 7: 583-600. doi.org/10.1007/s41748-023-00349-x

9. Haggblade S, Diarra A, Traoré A. Regulating agricultural intensification: Lessons from west Africa's rapidly growing pesticide markets. Dev Policy Rev. 2022; 40: e12545. doi.org/10.1111/dpr.12545

10. El-Sharkawy MA. Global warming: causes and impacts on agro-ecosystems productivity and food security with emphasis on cassava comparative advantage in the tropics/subtropics. Photosynthetica. 2014; 52: 161-178. doi.org/10.1007/s11099-014-0028-7

11. El-Sharkawy MA. Cassava biology and physiology. Plant Mol Biol. 2004; 56: 481-501. doi.org/10.1007/s11103-005-2270-7

12. Howeler RH. Long-term effect of cassava cultivation on soil productivity. Field Crops Res. 1991; 26: 1-18. doi.org/10.1016/0378-4290(91)90053-X

13. Howeler RH. Developing systainable cassava production systems with farmaers' involvement in Asia. In: Fujisaka S, editor. Systems and Farmaer Participatory Research. Developments in Research on Natural Resource Management. CIAT Publication; 1999. pp. 86-102.

14. Choenkwan S, Fox JM, Rambo AT. Agriculture in the mountains of northeastern Thailand: currebt situation and prospects for development. Mt Res Devl. 2014; 34: 95-106. doi.org/10.1659/MRD-JOURNAL-D-13-00121.1

15. Reynolds, TW, Waddington SR, Anderson CL, Chew A, True Z, Cullen A. Environmental impacts and constraints associated with the production of major food

crops in Sub-Saharan Africa and South Asia. Food Security. 2015; 7:795-822. doi.org/10.1007/s12571-015-0478-1

16. Delaquis E, de Haan S, Wyckhuys KAG. On-farm diversity offsets environmental pressures in tropical agro-ecosystems: a syntehtic review for cassava-based systems. Agric Ecosyst Environ. 2018; 251: 226-235. doi.org/10.1016/j.agee.2017.09.037

17. Iijima M, Izumi Y, Yuliadi E, Sunyoto S, Ardjasa WS. Cassa-based intercropping systems on Sumatra island in Indonesia: productivity, soil erosoion, and rooting zoon. Plant Prod Sci. 2004; 7: 347-355. doi.org/10.1626/pps.7.347

18. Oshunsanya O, Sammson VW, Yu H. Soil loss due to harvesting of peanut and cassava under traditional farming systems: Cost implicatios of soil nutrient loss. J Soil Water Conserv. 2022; 77: 240-248. doi.org/10.2489/jswc.2022.00111

19. Sumithra R, Thushyanthy M., Srivaratharasan T. Assesment of soil loss and nutrient depletion due to cassava harvesting: A case study from inout traditional agriculture. Int Soil Water Coserv Res. 2013; 1: 72-79. doi.org/10.1016/S2095-6339(15)30041-1

20. Kuhwald M, Busche F, Saggau P, Duttmann R. Is soil loss due to crop harvesting the most disregarded soil erosion process? A review of harvest erosion. Soil Tillage Res. 2022; 215: 105213. doi.org/10.1016/j.still.2021.105213

21. Howeler RH, Cadavid LF. Short- and long-term fertility traits in Colombia to determine the nutrient requirements of cassava. Nutr Cycl Agroecosystems. 1990; 26: 61-80. doi.org/10.1007/BF01048744

22. Chua MF, Youbee L, Oudthachit S, Khanthavong P, Veneklaas EJ, Malik AI. Pottassium fertilisation is required to sustain cassava yield and soil ferttility. Agronomy. 2020; 10: 1103. doi.org/10.3390/agronomy10081103

23. Gonçalves YS, Freitas MSM, de Carvalho AJC, Vieira ME, Peçanha DA, Cunha JM, et al. Potassium sources impact on cassava plant productivity, quality and mineral composition. J. Plant Nutr. 2022; 45: 86-94. doi.org/10.1080/01904167.2021.1949465

24. Putthacharoen S, Howeler RH, Jantawat S, Vichukit V. Nutrient uptake and soil erosion losses in cassava and six other crops in a Psamment in Eastern Thailand. Field Crops Res. 1998; 57: 113-126. doi.org/10.1016/S0378-4290(97)00119-6

25. Leitchm A, Anusontpornperm A, Thanachit S, Jindaluang W, Phun-Iam M. Cassava response to phosphorus fertilizer in Warin soil series amended with cassava tails and stalk-bontonite mixture. Trends Sci. 2023; 20: 4885. doi.org/10.48048/tis.2023.4885

26. Setyastika, US, Utami SR, Kurniawan S, Agustina C. Is long-term cassava-based agriculture sustainable? (Case study of potassium content in the soil). IOP Conf Ser: Earth Environ Sci. 2023; 1131: 012003. doi.org/10.1088/1755-1315/1131/1/012003

27. Nawata E, Nagata Y, Kono Y. Iwama K, Yamamoto T, Watanabe K, et al. Mapping of cassava productivity using GIS in Northeast Thailand. Jpn J Trop Agr. 2004; 48: 211-219. doi.org/10.11248/jsta1957.48.211

28. Phromma I, Pagdee A, Popradit A, Ishida A, Uttaranakorn S. Protected area co-management and land use conflicts adjacent to Phu Kao–Phu Phan Kham National Park, Thailand. J Sustain For. 2019; 38: 486-507. doi.org/10.1080/10549811.2019.1573689

29. Popradit A, Srisatit T, Kiratiprayoon S, Yohsimura J, Ishida A, Shiyomi M, et al. Anthropogenic effects on a tropical forest according to the distance from human settlements. Sci Rep. 2015; 5: 14689. doi.org/10.1038/srep14689

30. Ishida A, Yamaji K, Nakano T, Ladpala P, Popradit A, Yoshimura K, et al. Comparative physiology of canopy tree leaves in evergreen and deciduous forests in Thailand. Sci Data. 2023; 10: 601. doi.org/10.1038/s41597-023-02468-6

31. Burt, R. Soil Survey Laboratory Methods Manual. Soil Survey Labratory Investigations Report No. 42. USDA-NRCS; 2004.

32. Jensen JL, Schjønning P, Watts CW, Christensen BT, Munkholm LJ. Soil texture analysis revisited: Removal of organic matter matters more than ever. PLoS ONE. 2017; 12; e0178039. doi.org/10.1371/journal.pone.0178039

33. Soil Survey Staff. Keys to Soil Taxonomy. 12th Edition. USDA-NRCS; 2014.

34. Bremmer JM, Mulvaney CS. Total nitrogen. In: Page AL, Miller RH, Keeny DR, editors. Methods of Soil Analysis. Society of Agronomy and Soil Science Society of America; 1982. pp. 1119-1123.

35. Murphy J, Riley JP. A modified single solution method for the determination of phosphate in natural waters. Anal Chim.Acta. 1962; 27: 31-36. doi.org/10.1016/S0003-2670(00)88444-5

36. Jackson MLR, Barak P. Soil Chemical Analysis: Advanced Course. University of Wisconsin-Madison Libraries Papallel Press; 2005.

37. Mylavarapu R. Walkey-Black. Unit 5 Soil Organis Matter. In: Sikora FJ, Moore KP, editors. Soil Test Methods from the Southeastern United State. Southern Cooperative Series Bulletin No. 419. The Southern Association of Agricultural Experiment Station Directors; 2014. pp. 158-161. https://aesl.ces.uga.edu/Sera6/PUB/Methodsmanualfinalsera6.Pdf

38. Landon JR. Booker tropical soil manual: A handbook for soil survey and agricultural land evaluation in the tropics and subtropics. London, Routledge; 1991. https://doi.org/10.4324/9781315846842

39. Anastassiades M, Lehotay SJ, Štajnbaher D, Schenck FJ. Fast and easy multiresidue method employing acetonitrile extraction/partitioning and "Dispersive solid-phase extraction" for the determination of pesticide residues in produce. J AOAC Int. 2003; 86: 412-131. doi.org/10.1093/jaoac/86.2.412

40. R Development Core Team. 2019 R: A language and environmental for statistical computing. R Foundation for Statistical Computing. Available from https://https://www.R-project.org/

41. Ohta S, Morisada K, Tanaka N, Kiyono Y, Effendi S. Are soils in degradaded dipterocarp forest ecosystems deteriorated? A comparison of Imperata grasslands, degraded secondary forests, and primary forests. In: Guhardja E, Fatawi M, Sutisna M, Mori T, Ohta S, editors. Rainforest Ecosystems of East Kalimantan. Tokyo, Springer; 2000. pp 49-57.

42. Haynes RJ, Naidu R. Influence of lime, fertilizer and manure applications on soil organic matter content and soil physical conditions: a review. Nutr Cycl Agroecosyst. 1998; 51: 123-137. https://doi.org/10.1023/A:1009738307837

43. Jarecki MK, Lal Rattan, James R. Crop management effects on soil carbon sequestration on selected farms' fields in northeastern Ohio. Soil Tillage Res. 2005; 81: 265-276. https://doi.org/10.1016/j.still.2004.09.013

44. Kolawole OK, Awodum MA, Ojeniyi SO. Soil fertility improvement by *Tithonia diversifolia* (Hemsl.) A Gray and its effect on cassava performance and yield. Int J Eng Sci. 2014; 3: 36-43.

45. Bilong EG, Abossolo-Angue M, Ajebesone FN, Anaba BD, Madong BA, Nomo LB, et al. Improving spoil physical properties and cassava productivity through organic management in the southern Cameroon. Neliyon. 2022; 8: e09570. doi.org/10.1016/j.heliyon.2022.e09570

46. Fageria NK , Nascente AS. Management of soil acidity of South American soils for suitable crop production. Adv Agronomy. 2014; 128:221-275. doi.org/10.1016/B978-0-12-802139-2.00006-8

47. Punpom T, Leksungnoen P, Aramrak S, Kongsil P, Wisawapipat W. Triggering root proton efflux as an aluminum-detoxifying mechanism in cassava. Ann Agric Sci. 2022； 67: 173-180. https://doi.org/10.1016/j.aoas.2022.10.002

48. von Uexküll HR, Mutert E. Global extent, development and economic impact of acid soils. Plant Soil. 1995; 174: 1-15. doi.org/10.1007/BF00009558

49. Horst WJ , Wang Y, Etica D. The role of the root apoplast in aluminium-induced inhibition and in aluminium resistance of plants: a review. Ann Bot. 2010; 106: 185-197. doi.org/10.1093/aob/mcq053

50. Aune, JB, Lal R. Agricultural productivity in the tropics and critical limits of properties of Oxisols, Ultisols, and Alfisols. Trop Agric 1997; 74: 96-103. https://journals.sta.uwi.edu/ojs/index.php/ta/article/view/2488

51. Food and Agriculture Organization for the United Nations (FAO). Soil for nutrition: state of the art. Rome, FAO; 2022. https://https//www.fao.org/3/cc0900en/cc0900en.pdf

52. Dhaliwal SS, Narech RK, Mandal A, Singh R, Dhaliwai MK. Dynamics and transformations of micronutrients in agricultural soils as influenced by organic matter build-up: A review. Enviorn Sustaina Indic. 2019; 1-2; 100007. doi.org/10.1016/j.indic.2019.100007

53. Are KS, Oluwatasin GA, Adeyolanu OD, Oke AO. Slash and burn effect on soil quality of Alfispl : Soil physical properties. 2009; Soil Tillage Res. 103: 4-10. doi.org/10.1016/j.still.2008.08.011

54. Silva V, Montanarella L, Jones A, Fernández-Ugalde, Mol HGJ, Ritsema CJ, et al. Distributionof glyphosate and aminomethylphosphnic acid (AMPA) in agricultural topsoils of the European Union. 2018; Sci Total Environ. 62; 1352-1359. doi.org/10.1016/j.scitotenv.2017.10.093

55. Royal Gazette. Announcement of the Ministry of Agriculture and Cooperatives regarding discontinuing the use of labels and containers for hazardous substances related to glyphosate under the responsibility of the Department of Agriculture. 2019. (in Thai language) Available form: https://www.opsmoac.go.th/buengan-dwl-files-412891791918

56. Royal Gazette. Ministry of Industry Announcement Regarding the list of hazardous substances (6th edition). 2020. (in Thai language) Available form: https://www.ratchakitcha.soc.go.th/DATA/PDF/2563/E/117/T_0056.PDF

57. Royal Gazette. Regulations of the Department of National Parks, Wildlife and Plant Conservation on concerning on access to national parks. 2021. (in Thai language) Available form: https://portal.dnp.go.th/DNP/FileSystem/download?uuid=513724c5-98d9-4f18-a347-2799d1e20cea.pdf

58. Milesi MM, Lorenz V, Pacini G, Repetti, Demonte LD, Varayoud J, et al. Perinatal exposure to a glyphosate-based herbicide impaires female reproductive outcomes and induces second-generation adverse effects in Wistar rats. Arch. Toxicol. 2018; 93: 3635-3638. doi.org/10.1007/s00204-019-02609-0

59. Lorenz V, Milesi MM, Schimpf MG, Luque EH, Varayoud J. Epigenetic disruption of estrogen receptor alpha is induced by a glyphosate-based herbicide in the preimplantation uterus of rats. Mol Cell Endocrinol. 2019; 15: 133-141. doi.org/10.1016/j.mce.2018.120.022

60. Kusbad D, Nilsson EE, King SE, Sadler-Riggleman I, Beck D, Skinner MK. Assessment of glyphosare induced epigenetic transgenerational inheritance of pathlogies and sperm epimutations: generational toxicologt. Sci Rep 2019; 9: 6372. doi.org/10.1038/s41598-019-42860-0

61. Heap I. Global perspective of herbivide-resistant weeds. Pest Manag Sci. 2014; 70: 1306-1315. doi.org/10.1002/ps.3696

62. Squires CC, Coleman GRY, Broster JC, Preston C, Boutsalis P, Owen MJ, et al. Increasing the value and effciency of hebicide resistance surveys. Pest Manag Sci 2021; 77: 3881- 3889. doi.org/10.1002/ps.6333

63. Tahmasebi BK, Alebrahim MT, Roldán-Gómez RA, da Silveira HM, de Carvalho LB, la Cruz RA, et al. Effectiveness of alternative herbicides on three Conyza species from Europe with and without glyphosate resistance. Crop Prot. 2018; 112: 350–355. doi.org/10.1016/j.cropro.2018.06.021

64. Kanatas P, Anthonopoulos N, Gazoulis I, Travlos IS. Screening glyphosate alternative weed control options in important perennial crops. Weed Sci. 2021; 69: 704–718. doi.org/10.1017/wsc.2021.55

65. Garip F. The impact of migration and remittances on wealth accumulation and distribution in rural Thailand. Demograpy 2014; 51; 673-698. doi.org/10.1007/s13524-013-0260-y

66. Gutzler C, Helming K, Balla D, Dannowski R, Deumlich D. Glemnitz M, et al. Agricultural land use changes–a scenario-based sustainability impact assessment for Brandenburg, Germany. Ecol Indic. 2015; 48; 505-517. doi.org/10.1016/j.ecolind.2014.09.004

67. Chingprapa A, Maneeratana T, Dirdjosubroto W. Situation, needs and interests of rural young people: The case of Thailand. In: Report of the Inter-Country Worksjop on Population Education for the Rural Youth. Rome, DOAE-FAO; 1997. https://https://www.fao.org/3/X5630e/X5630e04.htm

68. Warr P. Poverty reduction through long-term growth: the Thai experience. Asian Econ Papers 2009; 8: 51-76. doi.org/10.1162/asep.2009.8.2.51

69. Kukla J, Whitfeld T, Cajthaml T, Baldrian P, Veselá-Šimáckovâ H, Novotný V, et al. The effect of traditional slash-and-burn agriculture on soil organic matter, nutrient content, and microbiota in tropical ecosystems of Papua New Guinea. Land Degrad Dev 2019; 30: 66-177. doi.org/10.1002/ldr.3203

70. Lal R, Ghuman BS, Shearer W. Cropping systems effects of a newly-cleared untisol in Southern Nigeria. Soi Technol. 1992; 5: 27-38. doi.org/10.1016/0933-3630(92)90004-K

71. Ardjasa WS, Ando H, Kimura M. Yield and soil erosion among sassava-baswed cropping patterns in South Sumatra. Soil Sci Plant Nutr. 2001; 47: 101-112. doi.org/10.1080/00380768.2001.10408372

72. Kuyper TW. Intercropping and crop rotations in cassava cultivation: a production systems approch. Cambride, Burleigh Dodds Cience Publishing Ltd; 2017.

73. Brenda BL. Resilience in agriclture through crop diversiication: Adaptive management for environmental change. BioScience 2011; 61: 183-193. doi.org/10.1525/bio.2011.61.3.4

74. Zabel F, Müller, Elliott J, Minoli S, Jägermeyr J, Schneider JM, et al. Large potential for crop production adaptation depends on available future varieties. Glob Chang Biol. 2021; 27: 3870-3882. doi.org/10.1111/gcb.15649

75. Horimoto S, Fukuda K, Yoshimura J, Ishida A. Fresh-marketable tomato yields enhanced by moderate weed control and supressed fruit dehiscence with woodchip mulching. Sci Rep. 2022; 12: 13283. doi.org/10.1038/s41598-022-15568-x

76. Hartwing NL, Ammon HU. Cover crops and living mulches. Weed Sci. 2002; 50: 688-699. doi.org/10.1614/0043-1745(2002)050[0688:AIACCA]2.0.CO;2

77. Samedani B, Ranjbar M, Rahimian H, Jahansoz MR. Utilization of rye and hairy vetch cover crops for weed control in transplanted tomato. Pak J Biol Sci. 2006; 9: 2323-2327. doi.org/10.3923/pjbs.2006.2323.2327

Figure and table legends

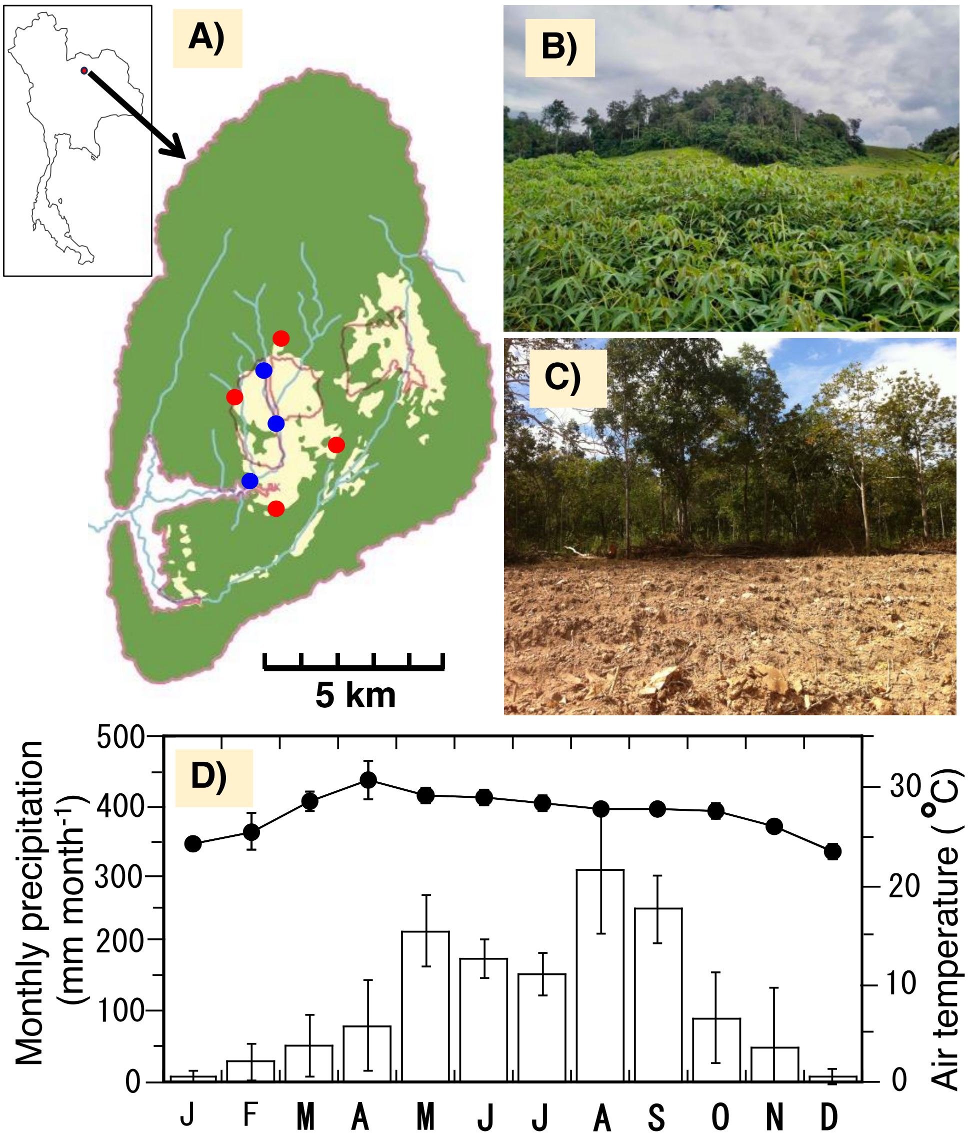

Fig 1. A map of Phu Koa, the photos of monocultural cassava farmland and the seasonal variations of precipitation and air temperature. (**A**) The maps of Phu Kao and Thailand. Green areas show natural forest and light-yellow areas show crop farmland (mainly by cassava) including human villages, and the light-blue lines show streams. The red lines indicate the boundary set by the Department of National Parks, Wildlife and Plant Conservation (DNP) in 2010, to legally separate the farmland and protected forest areas. Four red-points indicate the sampling sites of farmland and forest soil (north, east, south, and west of the main village), and three blue-points indicate those of stream water (upper, middle, and lower streams). The photos are the cassava farmland (**B**) before harvesting in the rainy season and (**C**) after harvesting in the dry season (by A. Popradit). (**D**) The monthly precipitation (bars) and the monthly averaged air temperature (closed cycles) during the research period (2016 to 2020). Vertical vars show ± 1 S.D.

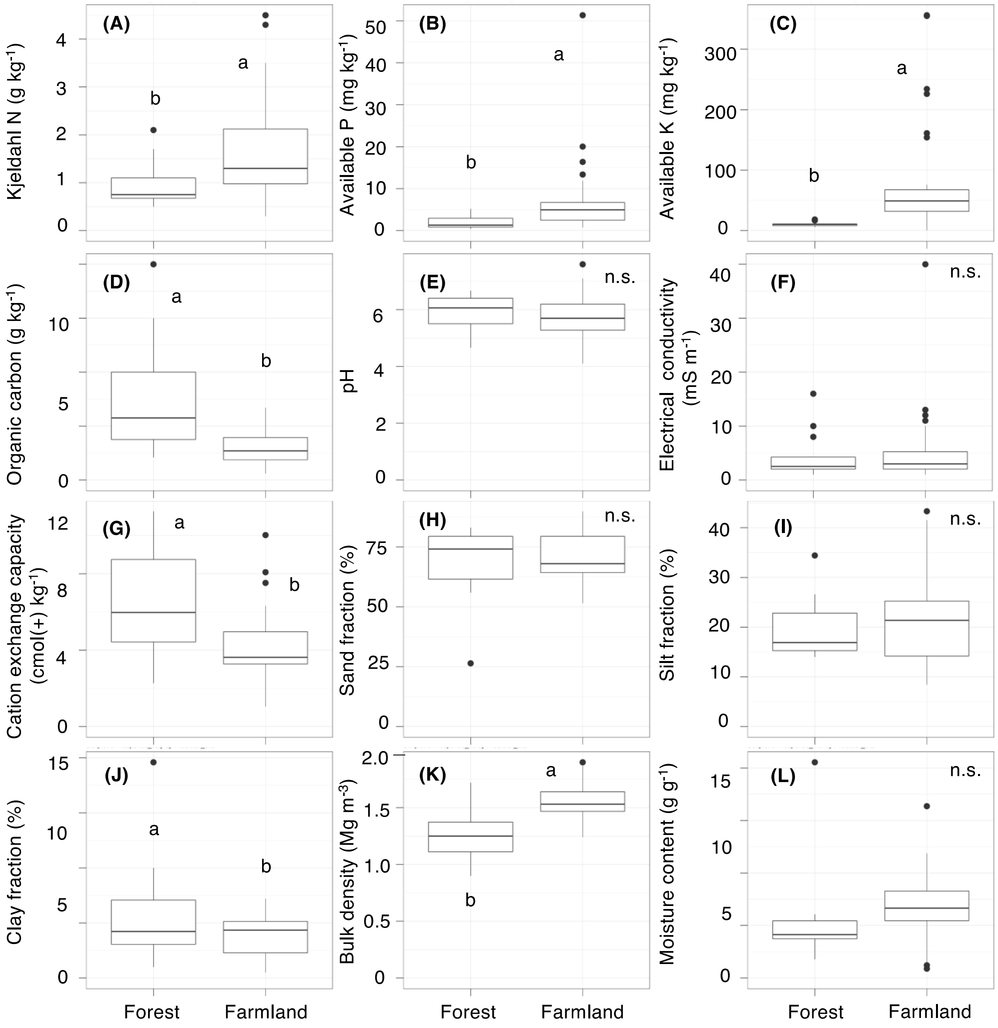

Fig 2. The soil properties of the farmland and its adjacent forest soils. (**A**) Kjeldahl nitrogen (the sum of organic N and ammonia N; g kg^{-1}), (**B**) available phosphorus (P; mg kg^{-1}), (**C**) available potassium (K; mg kg^{-1}), (**D**) organic carbon (g kg^{-1}), (**E**) pH, (**F**)

electrical conductivity (EC; mS m^{-1}), (**G**) cation exchange capacity (cmol(+) kg^{-1}), (**H**) fraction of sand (%), (**I**) fraction of silt (%), (**J**) fraction of clay (%), (**K**) bulk density (Mg m^{-3}), and (**L**) moisture content (g g^{-1}). The boxplot shows the median (horizontal bar), interquartile range (box), 5th and 95th percentiles (whiskers; vertical bars), and outliers (black closed points). Different letters (a, b, c) show the significant differences among the years, as follows: ***: $P < 0.001$, **: $P < 0.01$, *: $P < 0.05$, and n.s.: $P \geq 0.05$.

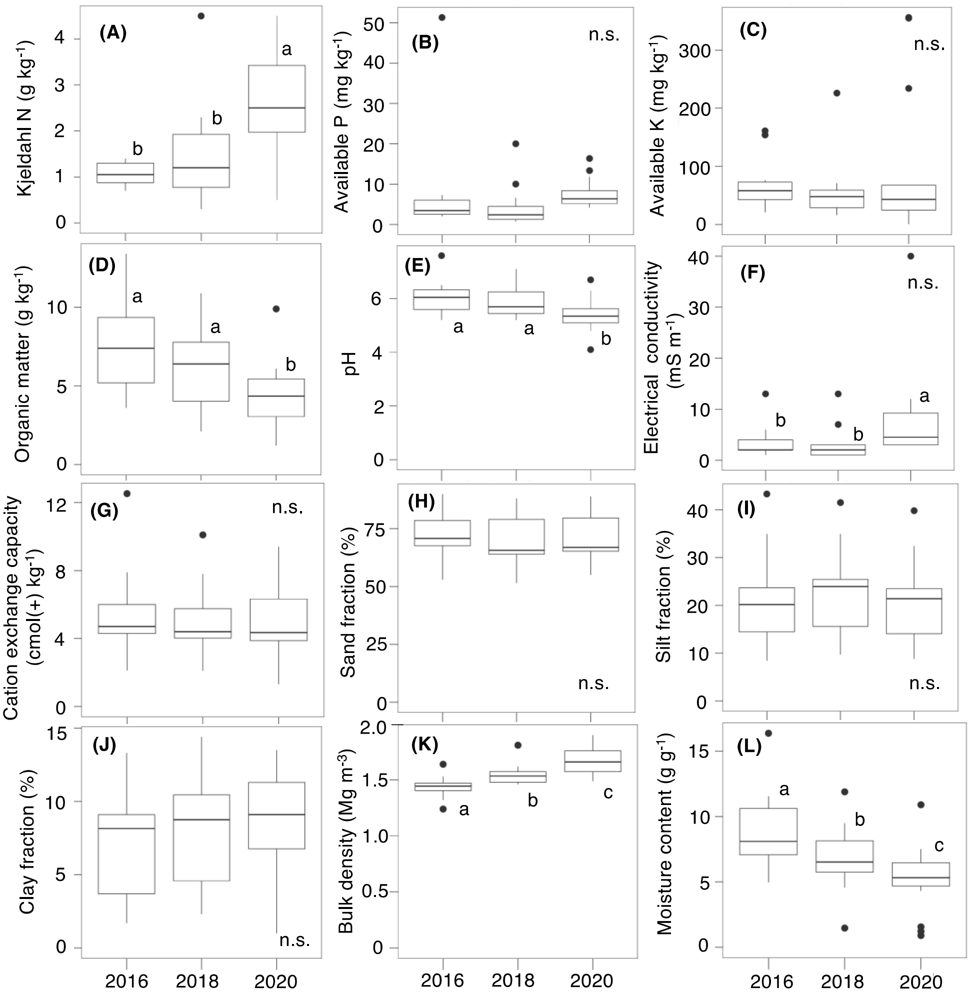

Fig 3. The yearly change in soil properties after crop harvesting in the dry season. (**A**) Kjeldahl nitrogen (the sum of organic N and ammonia N; g kg^{-1}), (**B**) available phosphorus (P; mg kg^{-1}), (**C**) available potassium (K; mg kg^{-1}), (**D**) organic carbon (g kg^{-1}), (**E**) pH, (**F**) electrical conductivity (EC; mS m^{-1}), (**G**) cation exchange capacity (cmol(+) kg^{-1}), (**H**) fraction of sand (%), (**I**) fraction of silt (%), (**J**) fraction of clay (%),

(**K**) bulk density (Mg m^{-3}), and (**L**) moisture content (g g^{-1}). The boxplot shows the median (horizontal bar), interquartile range (box), 5th and 95th percentiles (whiskers; vertical bars), and outliers (black closed points). Different letters (a, b, c) show the significant differences among the years, as follows: ***: $P < 0.001$, **: $P < 0.01$, *: $P < 0.05$, and n.s.: $P \geq 0.05$.

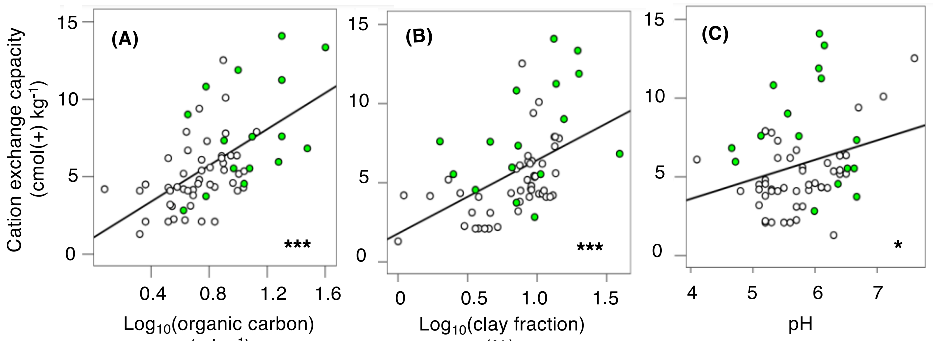

Fig 4. Relationships of the cation exchange capacity against (A) $\log_{10}$(organic carbon), (B) $\log_{10}$(the fraction of clay), and (C) pH. Open circles represent data in the

farmland soils, and closed green circles represent data in the forest soils. ***: P < 0.001, *: P < 0.05.

Table 1. Soil properties of the farmland and the natural forest. The mean (1 S.D.) values are shown for each property. The significance differences between the sites based on one-way ANOVA are shown as follows; ***: $P < 0.001$, **: $P < 0.01$, *: $P < 0.05$, and n.s.: $P \geq 0.05$.

Soil properties	unit	Farmland soil	Forest soil	df	Sum Squre	Mean Square	F value	P value	Significance
Kjeldahl nitrogen (N)	g kg^{-1}	1.7 (1.1)	0.9 (0.5)	1	7.02	7.02	7.58	**0.0077**	**
Avalable phosphorus (P)	mg kg^{-1}	6.2 (7.7)	2.0 (1.5)	1	218.69	218.69	4.77	**0.0328**	*
Avalable posassium (K)	mg kg^{-1}	68.8 (76.8)	11 (3.8)	1	40736.56	40736.56	9.11	**0.0037**	**
Organic matter	g kg^{-1}	5.9 (2.7)	14.5 (0.99)	1	896.57	896.57	30.60	**0.0000**	***
pH		5.7 (0.6)	5.9 (0.7)	1	0.31	0.31	0.76	0.3859	n.s.
Electrical conductivity (EC)	mS m^{-1}	5 (6.1)	4 (4.3)	1	3.52	3.52	0.11	0.7443	n.s.
Cation exchange capacity	cmol (+) kg^{-1}	5.7 (2.2)	8.0 (3.4)	1	105.04	105.04	16.11	**0.0002**	*
Sand fraction	%	70.5 (10.4)	69.3 (14.6)	1	18.75	18.75	0.14	0.7085	n.s.
Silt fraction	%	21.5 (8.80)	19.3 (5.7)	1	57.31	57.31	0.86	0.3565	n.s.
Clay fraction	%	8.0 (3.63)	11.4 (8.3)	1	141.63	141.63	4.59	**0.0362**	*
Bulk density	Mg m^{-3}	1.56 (0.141)	1.24 (0.214)	1	1.19	1.19	45.49	**0.0000**	***
Moisture content	g g^{-1}	6.98 (2.909)	5.20 (4.257)	1	37.92	37.92	3.51	0.0657	n.s.

Table 2. The statistical differences among the five years (2016-2020) and among the four locations in each soil property, using repeated one-way ANOVA.

Significances are shown as follows, ***: P < 0.001, **: P < 0.01, and *: P < 0.05.

Soil properties	Unit	Expranatory variable	df	Sum Squre	Mean Square	F values	P values	Significance
Kjeldahl nitrogen (N)	g kg^{-1}	Year	2	19.99	10.00	13.93	**0.000**	***
		Location	3	4.10	1.37	1.90	0.144	n.s
Avalable phosphorus (P)	mg kg^{-1}	Year	2	114.86	57.43	1.00	0.379	n.s
		Location	3	149.88	49.96	0.87	0.482	n.s
Avalable posassium (K)	mg kg^{-1}	Year	2	8894.20	4447.10	1.10	0.347	n.s
		Location	3	8034.00	2678.00	0.66	0.592	n.s
Organic carbon	g kg^{-1}	Year	2	78.71	39.35	24.98	**0.000**	***
		Location	3	26.27	8.76	5.56	**0.013**	*
pH		Year	2	3.15	1.58	11.97	**0.000**	***
		Location	3	0.45	0.15	1.15	0.370	n.s
Electrical conductivity (EC)	mS m^{-1}	Year	2	241.29	120.65	4.04	**0.025**	*
		Location	3	257.73	85.91	2.88	**0.047**	*
Cation exchange capacity	cmol (+) kg^{-1}	Year	2	1.00	0.50	1.28	0.294	n.s
		Location	3	0.51	0.17	0.44	0.730	n.s
Sand fraction	%	Year	2	52.32	26.16	3.96	**0.030**	*
		Location	3	41.69	13.90	2.10	0.153	n.s
Silt fraction	%	Year	2	20.76	10.38	4.98	**0.014**	*
		Location	3	9.42	3.14	1.51	0.263	n.s
Clay fraction	%	Year	2	19.98	9.99	2.82	0.075	n.s
		Location	3	11.57	3.86	1.09	0.391	n.s
Bulk density	Mg m^{-3}	Year	2	0.48	0.24	46.21	**0.000**	***
		Location	3	0.03	0.01	1.90	0.184	n.s
Moisture content	g g^{-1}	Year	2	103.03	51.52	15.93	**0.000**	***
		Location	3	17.92	5.97	1.85	0.192	n.s

Table 3. The results of multiple comparisons (Tukey's honestly significant difference test) among the years and locations. The statistical comparisons were conducted only in soil properties with significant differences ($P < 0.05$) in Table 3. 2016, 2018, and 2020 are the examined years. N: north, E: east, W: west, S: south locations. ***: $P < 0.001$, **: $P < 0.01$, *: $P < 0.05$, and n.s.: $P \geq 0.05$.

Soil properties	Unit	Expranatory variables		Estimated	1 S.D.	*T* values	*P* values	Significance
Kjeldahl N	g kg^{-1}	Year	2018-2016	0.3375	0.299551	1.127	0.835711	n.s.
			2020-2016	1.50625	0.299551	5.028	**0.0001**	***
			2020-2018	1.16875	0.299551	3.902	**0.000822**	***
Organinc carbon	g kg^{-1}	Location	N-E	1.9083	1.1467	1.664	0.4868	n.s.
			S-E	-2.7083	1.1467	-2.362	0.1283	n.s.
			W-E	-0.7833	1.1467	-0.683	0.9781	n.s.
			S-N	-4.6167	1.1467	-4.026	**0.001**	***
			W-N	-2.6917	1.1467	-2.347	0.1328	n.s.
			W-S	1.925	1.1467	1.679	0.4769	n.s.
		Year	2018-2016	-1.4062	0.4437	-3.169	0.0127	*
			2020-2016	-3.1312	0.4437	-7.056	**0.001**	***
			2020-2018	-1.725	0.4437	-3.887	**0.001**	***
pH		Year	2018-2016	-0.1625	0.1283	-1.267	0.75629	n.s.
			2020-2016	-0.6063	0.1283	-4.726	**0.0001**	***
			2020-2018	-0.4438	0.1283	-3.46	**0.00463**	**
EC	mS m^{-1}	Location	N-E	2.9167	2.2298	1.308	0.7305	n.s.
			S-E	-1	2.2298	-0.448	0.9968	n.s.
			W-E	4.8333	2.2298	2.168	0.199	n.s.
			S-N	-3.9167	2.2298	-1.757	0.4247	n.s.
			W-N	1.9167	2.2298	0.86	0.9417	n.s.
			W-S	5.8333	2.2298	2.616	0.0673	n.s.
		Year	2018-2016	-0.6875	1.9311	-0.356	0.999	n.s.
			2020-2016	4.375	1.9311	2.266	0.1604	n.s.
			2020-2018	5.0625	1.9311	2.622	0.0662	n.s.
Sand	%	Year	2018-2016	-2.5563	0.9085	-2.814	**0.0385**	*
			2020-2016	-1.2125	0.9085	-1.335	0.7134	n.s.
			2020-2018	1.3438	0.9085	1.479	0.6156	n.s.
Silt	%	Year	2018-2016	1.2687	0.5104	2.486	0.0946	n.s.
			2020-2016	-0.225	0.5104	-0.441	0.9971	n.s.
			2020-2018	-1.4938	0.5104	-2.927	0.0275	*
Bulk Density	Mg m^{-3}	Year	2018-2016	0.11062	0.02539	4.357	**0.000129**	***
			2020-2016	0.24375	0.02539	9.6	**0.0001**	***
			2020-2018	0.13312	0.02539	5.243	**0.0001**	***
Moisture content	g g^{-1}	Year	2018-2016	-1.9744	0.6358	-3.105	**0.0155**	*
			2020-2016	-3.5825	0.6358	-5.635	**0.001**	***
			2020-2018	-1.6081	0.6358	-2.529	0.0845	n.s.

Table 4. The results of multiple regression analysis for the cation exchange capacity. The cation exchange capacity is the response (dependent) variable, and $\log_{10}$(organic carbon), $\log_{10}$(clay fraction), and pH are explanatory variables. "Estimated" means the slope for each variable. ***: $P < 0.001$, **: $P < 0.01$, *: $P < 0.05$.

Responsible variable	Expranatory variables	Estimated	1 S.D.	*T* values	*P* values	Significance
Cation exchange capacity	Intercept	-6.061	2.6758	-2.265	**0.027129**	*
	log_{10}(organic carbon)	3.9304	1.1047	3.558	**0.000738**	***
	log_{10}(clay fraction)	3.1796	1.0334	3.077	**0.00315**	**
	pH	1.0267	0.4426	2.32	**0.023765**	*

Table 5. The concentrations of herbicides (paraquat and glyphosate) in the stream water (upper, middle, and lower streams) and the farmland soil. The herbicide concentrations were examined in the rainy and dry seasons in 2020. The units of the concentrations in stream water and soil are mg l^{-1} (ppm) and mg kg^{-1} (ppm), respectively. "n.d." means "not detected".

	Paraquat		Glyphosate	
	Dry season	Rainny season	Dry season	Rainny season
Upper stream	n.d.	n.d.	n.d.	n.d.
Middle stream	n.d.	n.d.	**0.5**	n.d.
Lower stream	n.d.	n.d.	n.d.	n.d.
North_Subplot 1	n.d.	n.d.	n.d.	**1.5**
North_Subplot 2	n.d.	n.d.	**1.3**	n.d.
North_Subplot 3	n.d.	n.d.	n.d.	**5.0**
North_Subplot 4	n.d.	n.d.	n.d.	n.d.
East_Subplot 5	n.d.	n.d.	n.d.	**1.5**
East_Subplot 6	n.d.	n.d.	n.d.	n.d.
East_Subplot 7	n.d.	n.d.	n.d.	n.d.
East_Subplot 8	n.d.	n.d.	n.d.	n.d.
West_Subplot 9	n.d.	n.d.	**1.3**	n.d.
West_Subplot 10	n.d.	n.d.	n.d.	**1.5**
West_Subplot 11	n.d.	n.d.	n.d.	n.d.
West_Subplot 12	n.d.	n.d.	n.d.	n.d.
South_Subplot 13	n.d.	n.d.	n.d.	n.d.
South_Subplot 14	n.d.	n.d.	n.d.	**2.5**
South_Subplot 15	n.d.	n.d.	**2.5**	n.d.
South_Subplot 16	n.d.	n.d.	n.d.	n.d.
