# Supplementary material for: Soil degradation and herbicide pollution by repeated cassava monoculture within Thailand’s conservation region
Source: PLoS One. 2024 Aug 6;19(8):e0308284. doi: 10.1371/journal.pone.0308284 (PMC11302848; doi:10.1371/journal.pone.0308284)
Supplement: Attachment — Submitted filename: Comments_PONE-D-24-04738-R2.pdf [file pone.0308284.s007.pdf]

General comments

The manuscript has been significantly improved. Though the quality of the writeup is still low, it could be considered for publication. The authors are, however, advised to address the following specific comments:

Lines 50 – 51: Revise the sentence. It could read as “We hypothesise that long-term cassava monoculture leads to the degradation of soil properties”.

Line 111: Replace “harvesting” with “harvested”

Line 126: Long-term

Lines2 215 – 217: The sentence is incomprehensible. Revise it.

Line 308: Delete “not”

Lines 337 and 368: Replace “has” with “is”

Line 369: …leads to…

Lines 379 – 380: “the averaged pH **leached** to”?
