# Supplementary material for: Soil degradation and herbicide pollution by repeated cassava monoculture within Thailand’s conservation region
Source: PLoS One. 2024 Aug 6;19(8):e0308284. doi: 10.1371/journal.pone.0308284 (PMC11302848; doi:10.1371/journal.pone.0308284)
Supplement: Attachment — Submitted filename: Response to Reviewers.docx [file pone.0308284.s008.docx]

PONE-D-24-04738R2

Soil degradation and herbicide pollution by repeated cassava monoculture within Thailand’s conservation region

Dear Dr. Ishida,

Thank you for submitting your manuscript to PLOS ONE. After careful consideration, we feel that it has merit but does not fully meet PLOS ONE’s publication criteria as it currently stands. Therefore, we invite you to submit a revised version of the manuscript that addresses the points raised during the review process.

Reply: Thank you for your support for publishing in PLoS ONE. The previous MS included some parts of mistyping English. According to the comments of Reviewer 1, we revised all parts.

Reviewer #1: General comments

The manuscript has been significantly improved. Though the quality of the writeup is still low, it could be considered for publication. The authors are, however, advised to address the following specific comments:
Reply: Thank you for your support for publishing in PLoS ONE. The previous MS included some parts of mistyping English. According to your comments, we revised all parts, as follows：

Lines 50 – 51: Revise the sentence. It could read as “We hypothesise that long-term cassava monoculture leads to the degradation of soil properties”.

Reply：　We added the terms of “the degradation of”. (Line 51 in the revised MS)

Line 111: Replace “harvesting” with “harvested”

Reply：　We replace to “harvested”. (Line 111 in the revised MS)

Line 126: Long-term

Reply：　We revised it. (Line 126 in the revised MS)

Lines 215 – 217: The sentence is incomprehensible. Revise it.
Reply：　We revised this part, as follows:” The fresh mass of soil within the inner cylinder (242 cm³) of the drop-hammer sampler was measured using an electronic balance. The soil samples were then dried in an oven and subsequently placed in a desiccator to cool down to room temperature.” (Line 216-219 in the revised MS)

Line 308: Delete “not”

Reply：　We deleted “not”. (Line 308 in the revised MS)

Lines 337 and 368: Replace “has” with “is”

Reply：　We replaced to “is”. (Line 368 in the revised MS)

Line 369: …leads to…

Reply：　We added “to”. (Line 368 in the revised MS)

Lines 379 – 380: “the averaged pH leached to”?
Reply：　We revised this part, as follows:” the values of pH decreased to 4.3 to 4.4 in Indonesia [57, 58] and to 4.6 in Nigeria [59].” (Line 380-381 in the revised MS)

--------------------

Reviewer #2: All comments sent to the article's authors have been corrected. There are no additional comments.

Reply：　Thank you for your support for publishing in PLoS ONE.
